# Comprehensive analysis of structural and sequencing data reveals almost unconstrained chain pairing in TCRαβ complex

**DOI:** 10.1371/journal.pcbi.1007714

**Published:** 2020-03-12

**Authors:** Dmitrii S. Shcherbinin, Vlad A. Belousov, Mikhail Shugay

**Affiliations:** 1 Shemyakin and Ovchinnikov Institute of Bioorganic Chemistry, Moscow, Russia; 2 Pirogov Russian Medical State University, Moscow, Russia; 3 Center of Life Sciences, Skolkovo Institute of Science and Technology, Moscow, Russia; Fred Hutchinson Cancer Research Center, UNITED STATES

## Abstract

Antigen recognition by T-cells is guided by the T-cell receptor (TCR) heterodimer formed by α and β chains. A huge diversity of TCR sequences should be maintained by the immune system in order to be able to mount an effective response towards foreign pathogens, so, due to cooperative binding of α and β chains to the pathogen, any constraints on chain pairing can have a profound effect on immune repertoire structure, diversity and antigen specificity. By integrating available structural data and paired chain sequencing results we were able to show that there are almost no constraints on pairing in TCRαβ complexes, allowing naive T-cell repertoire to reach the highest possible diversity. Additional analysis reveals that the specific choice of contacting amino acids can still have a profound effect on complex conformation. Moreover, antigen-driven selection can distort the uniform landscape of chain pairing, while small, yet significant, differences in the pairing can be attributed to various specialized T-cell subsets such as MAIT and iNKT T-cells, as well as other TCR sets specific to certain antigens.

## Author summary

In the present paper we study chain pairing preferences in the T-cell receptor (TCR) heterodimer complex. The TCR molecule is formed by α and β chains and binding of both of these chains to an antigen presented by the major histocompatibility complex (MHC) molecule is required in order to trigger an immune response against foreign pathogens and neoantigens. We show that chain pairing in the TCR complex is nearly random ensuring a highly diverse set of TCRs required for recognition of a vast set of antigens. Our results also show that chain pairing preferences can nevertheless influence TCR complex geometry and biases in TCR chain pairing can be used to identify antigen-driven selection or selection towards specialized subsets of T-cells such as mucosal-associated and natural killer invariant T-cells.

## Introduction

The process of somatic recombination can produce an immense diverse repertoire of TCR α and β chain sequences in human, having a theoretical bound on the number of unique variants of ~10^21^ for TCR β chain alone [1]. The effective T-cell diversity is thus only limited by the total number of T-cells in human that is ~10^11^ [1] and potential pairing preferences between α and β chains. The size of foreign peptide pool to be recognized by T-cells can reach ~10^12^ variants for all possible 9-mers presented by HLA class I. So, as both TCR α and β chain is required to recognize an antigen [2] and a certain degree of cross-reactivity is needed to be able to form an efficient immune response [3], one would expect the immune system to aim at producing the highest possible number of α and β chain combinations across distinct T-cells in order to ensure optimal recognition of newly encountered pathogens.

Early estimates of the extent of pairing in human TCRαβ complex [4] indicate that on average, around 25 distinct α chains can be observed to pair with the same β chain in different T-cell clones at the level of individual T-cell repertoires. However, given the limited amount of unique TCRαβ clones that can be obtained via combinatorial single-chain high-throughput sequencing methods (~10^4−5^ in PairSEQ datasets [5] or by frequency-based pairing [6]) and even smaller typical yield of single-cell methods (~10^3−4^ cells according to 10x Genomics dataset compendium [7]), it is nearly impossible to directly quantify and enumerate the range of possible αβ pairings as recapturing the same α or β chain sequence is highly unlikely event for naive T-cells. The latter suggests that the exploration of pairing preferences should be performed indirectly by using statistical models to extrapolate consistent patterns observed in available TCRαβ repertoire data. For example, a recent study [8] uses statistical modelling to show that there are certain subtle (yet significant) biases in αβ pairing at the nucleotide level that stem from the genome organization of α and β loci and intrinsic biases of the V(D)J rearrangement process. Certain biases of αβ pairing were also reported to be related to CD4/CD8 T-cell differentiation [9].

In the present study, we utilize structural data to infer the set of contacting residues between α and β chains that should convey all pairing information in the absence of antigen-driven selection. We deliberately exclude the middle portion of CDR3 sequences that are involved in antigen recognition and are therefore subject to a multitude of selection biases. As amino acids at certain contacting positions are strongly linked, we operate on the level of individual residues instead of V/J genes and use Bayesian network approach to infer direct inter-chain correlations separating them from intra-chain correlations.

We report a conserved pattern of TCRαβ contacts between human and mouse, some of which involve highly conserved residues so that no information can be extracted from them. Frequentist inference of pairing preferences obtained from high-throughput sequencing data revealed certain residues that are paired significantly more often than expected by chance. Bayesian network analysis, however, shows that these residues are rarely encountered and there is insufficient information for building an accurate αβ pairing predictor, leading to a conclusion that the pairing is almost random. Finally, deeper analysis of available datasets suggests that pairing preferences can be heavily altered by antigen-driven selection and some αβ chain interactions encode invariant T-cell subsets.

## Results

### Contact maps of TCRαβ complexes

We started our analysis with exploring contact frequencies of α and β chain residues of known TCR:peptide:MHC complexes available via the PDB database for both human and mouse. Next, we proofread and corrected the complexes and mapped Variable (V) and Joining (J) gene sequences to produce complementarity determining region (CDR)/framework region (FR) markup and enumerate the residues of TCR chains (see **Materials and Methods** section).

We utilized IMGT numbering [10] to index residues and bring all sequences to a uniform coordinate set. Additionally, we discarded the middle part of the CDR3 region as it is mostly involved in TCR:antigen recognition and forms an extremely flexible omega loop that doesn't allow meaningful indexing across distinct TCRs.

**Fig 1:**
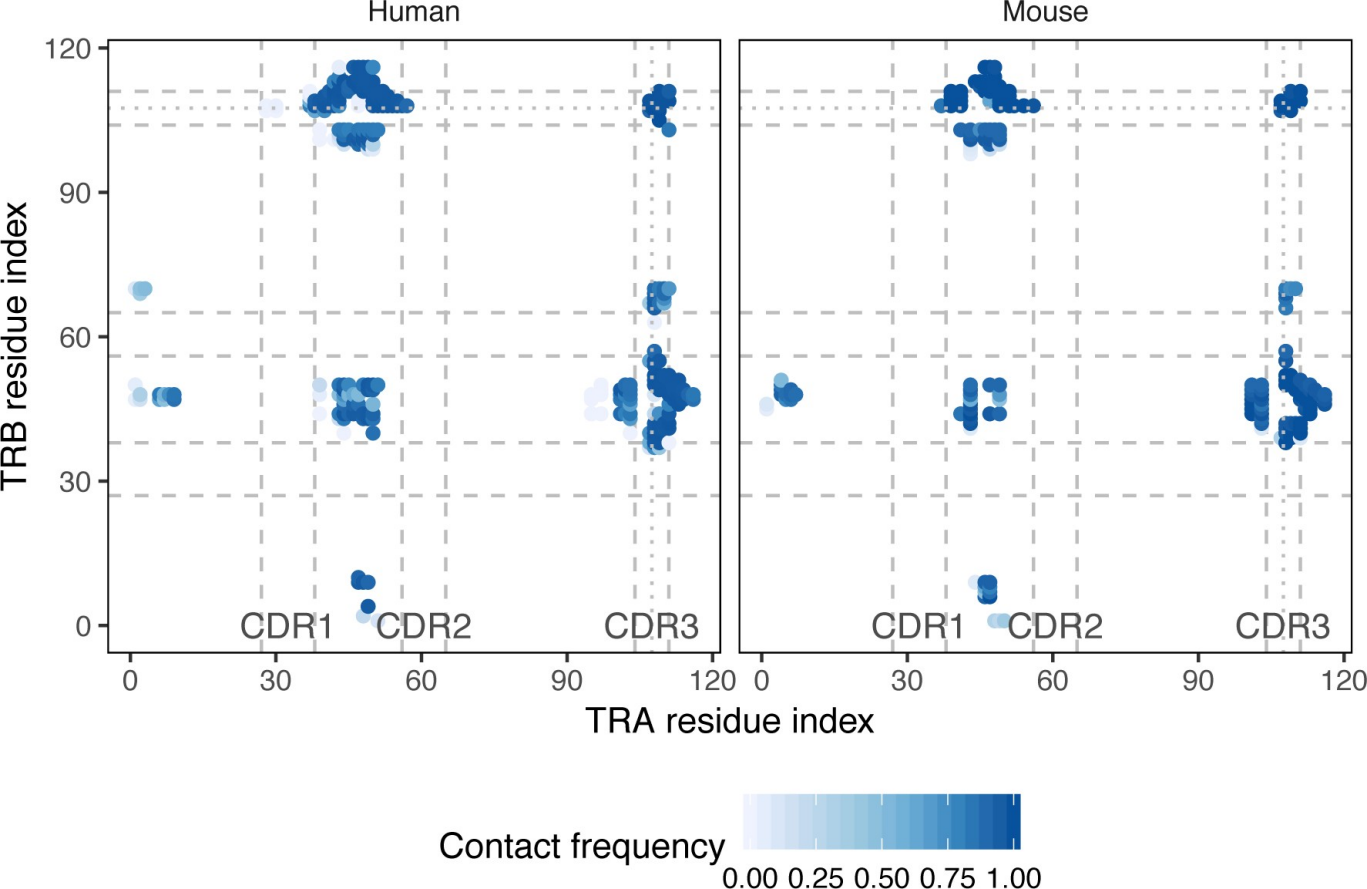
Contact map of TCRαβ complexes. A heatmap of inter-chain residue contact frequencies observed in *n = 131* human and *n = 39* mouse TCR:peptide:MHC complexes. Residue pairs having a distance between closest atoms of less than 5Å in at least one complex were considered in the analysis. Contact frequency was estimated by counting the number of times a given residue pair has a C_α_ distance of less than 15Å in PDB structures. CDR regions are shown with dashed lines, excluded middle portion of CDR3 is shown with dotted line.

The contact residues were defined based on C_α_ atom distance in order to compensate for the presence of distinct side chains that may not be fully covered by the available structural data (see **Materials and Methods** section). Resulting contact maps (**Fig 1**) show conservation between human and mouse and were highly symmetric: they feature contacts between FR1 and FR2 regions, FR2:FR2 contacts, FR2:CDR3 flank contacts and contacts between CDR3 flanks of different chains. There also is a visible non-symmetric contact region between the start of FR3 of β chain and J part of CDR3 of α chain.

### Sequence variability at contacting residues

**Fig 2:**
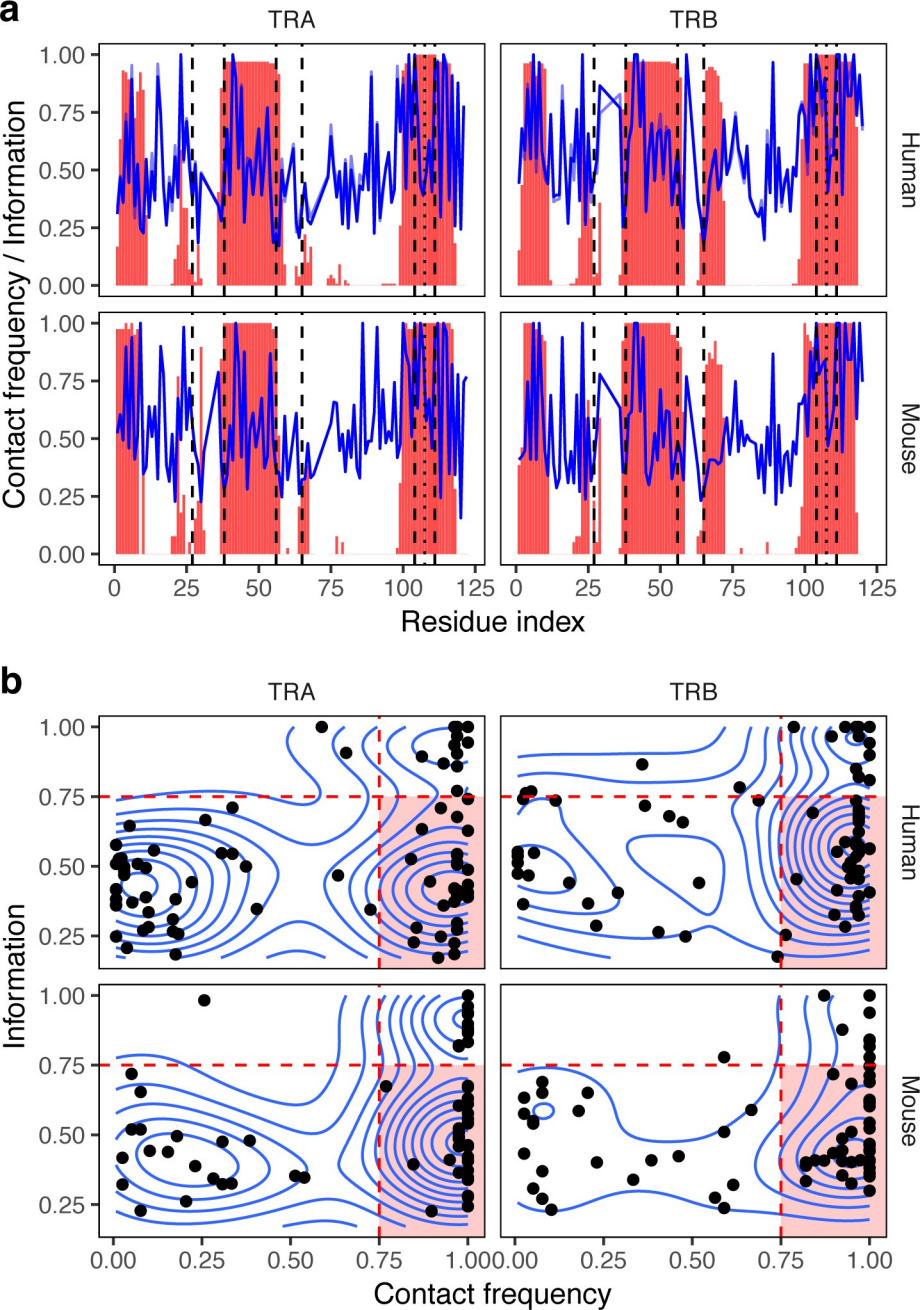
Variability and contact frequency of TCR chain residues. **a.** Contact frequency (red bars) and information content (blue line) across α and β chains. For human, transparent blue line shows information content computed from residue frequencies weighted by corresponding V and J gene usage in donor repertoires. Dashed lines show FR-CDR boundaries. **b.** Scatter plot represents the probability of being involved in inter-chain contact (x axis) and information content of amino acid frequency distribution (y axis) for individual α and β chain residues. Region with a selection of frequently contacted residues (*x > 0*.*75*) that are have variable amino acid content (*y < 0*.*75*) is highlighted with a red background. Residues with more information content should be considered as less variable, residues having no inter-chain contacts are not shown.

We further explored the amino acid content of various positions of V and J genes of α and β chains and compared the variability of amino acid content for a given residue to the frequency of inter-chain contacts in which it was involved (**Fig 2**). We quantified amino acid content variability by estimating the information content (computed as one minus normalized entropy for the vector of amino acid probabilities, see Methods) from the position-weight matrix of all V and J genes aligned according to IMGT. Our results show that contact residues can occur both in highly variable regions, as well as regions conserved across genes; the same holds true for weighted information scores that account for differential V and J usage in observed repertoires (**Fig 2A**).

While there is a small correlation between residue variability and contact frequency (R = 0.2, P < 0.05 for TRA, R = 0.3, P < 0.01 for TRB, in both human and mouse), scatter plot of these two variables for various inter-chain contact positions show the presence of three groups of residues for both chains in human and mouse (**Fig 2B**). Almost all contacting positions that are not frequently contacted have variable amino acid content, on the other hand, there are two groups of residues involved in inter-chain contacts with high and low variability of amino acid profile. In our further analysis we focused only on the latter (highlighted area in **Fig 2B**), as they are both involved in direct inter-chain contacts and have enough variability in their amino acid content required to study chain pairing preferences.

### Statistics of amino acid pairing preferences at contact residues

In order to study the (non)randomness of chain pairing in TCRαβ complex we used a large dataset of TCR clones reported in the PairSEQ study [5] (see **Materials and Methods** section). We calculated the number of times a given amino acid pair is observed at a given contact (residue pair) for the set of residues selected as described in the previous section. We next normalized the resulting amino acid frequency matrices for each residue pair to calculate expected counts under the hypothesis of random amino acid pairing at a given contact.

**Fig 3:**
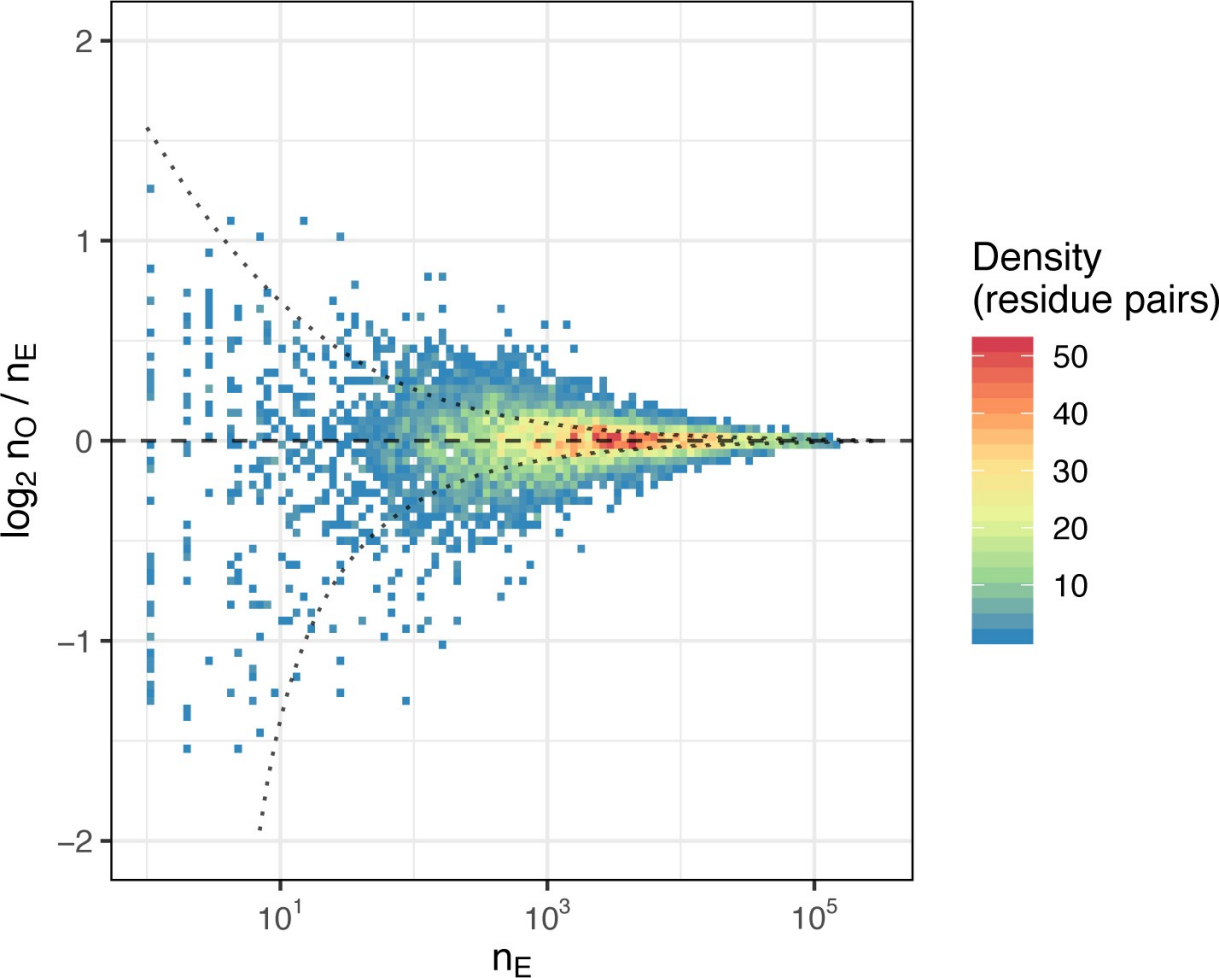
Amino acid statistics at contacting residues. A two dimensional density plot comparing the number of times a given amino acid combination is observed at an inter-chain contact versus the number of times it was expected to be observed n_E_. The expected count n_E_ is calculated using amino acid frequency distributions at separate chains and assuming random amino acid pairing; higher n_O_ / n_E_ ratio suggests enrichment of a given amino acid pair at corresponding contacting residues. The number of contacting residue pairs observed with certain n_O_ and n_E_ values (density of points at a given bin) is highlighted by color. Dotted lines show 95% confidence interval for the n_O_ / n_E_ ratio assuming Normal distribution with standard deviation computed from Binomial distribution approximated by n_E_ and the total number of observations n_T_ ≈ 3x10^5^.

The logarithm of the ratio of observed to expected counts of amino acid pairs is plotted in **Fig 3**. Ratios higher than 2.0 or less than 0.5 are only present for rare amino acid pairs with frequencies less than 10^3^ out of ~10^5^ total observations, in line with the growing variance of the ratio for smaller counts. The latter suggests that there is little, if any, pairing preference and this pairing preference is limited to rare TCR clones.

We computed Chi-squared and mutual information (MI) values for amino acid frequency matrices, the relation between MI and both the conservation of contacting residues and contact frequency for a given residue pair. As can be seen in **Fig Aa in S1 Appendix**, Chi-squared and MI values are in perfect agreement, yet one can note that the absolute value of MI < 0.003, meaning that while there are highly significant associations between contacting amino acids, the information carried by them is relatively small in agreement with observations from the previous paragraph. Highest MI scores are reached for the residue pairs with least conservation (**Fig Ab in S1 Appendix**). There is little correlation between MI and residue pair contact frequency (**Fig Ac in S1 Appendix**), suggesting the presence of indirect correlations.

An example of such indirect correlation between amino acid profiles is given in **Fig Ba in S1 Appendix**, where the top 5 α chain residues that contact with β_101_ by their MI score were listed. Notably, residues α_47_ and α_43_ have similar MI scores yet the frequency of their contacts with β_101_ is substantially different, 95% and 11% respectively. As can be seen from amino acid frequency matrices in **Fig Bb in S1 Appendix**, the amino acid preference profile is nearly the same for Tryptophan of α_47_ and Histidine of α_43_. On the other hand, there is only a single V gene that had both amino acids at given positions, as highlighted in **Fig Bc in S1 Appendix**. Thus, one is able to assume that Tryptophan at α_47_ is directly contacted by β_101_ based on the contact frequency difference, while the high score of Histidine of α_43_ is merely an artifact arising from the linkage of α_47_ and α_43_ in a single V gene.

### Bayesian network analysis reveals that pairing in TCRαβ complexes is almost random

In order to assess the actual information carried in inter-chain residue contacts that is relevant to TCRαβ chain pairing preferences we built a Bayes network (BN, see **Materials and Methods** section) of amino acid contact frequencies at residues that are frequently in contact between chains, as highlighted in **Fig Bb in S1 Appendix**. BN also helped us to resolve indirect correlations shown in **Fig Bc in S1 Appendix** and discussed in the previous section. Apart from allowing us to tell direct correlations of amino acid content at contacting residues from indirect ones, BN provides a factorization for the amino acid pairing probability distribution at contacting residues allowing us to estimate the amount of information conveyed by direct inter-chain contacts.

The BN construction for the entire PairSEQ dataset with no restriction of edges resulted in the structure shown in **Fig C in S1 Appendix**. In this case, a simple Hill-Climbing algorithm was used with a metric to optimize being Akaike Information Criterion (AIC, used in all BN learning throughout this section). Notably, only two edges connecting α and β components were observed: β_101_→α_43_ and α_46_→β_10_. The latter suggests that there is little information in inter-chain pairing, in line with the previous observations.

We therefore built a BN with only inter-chain contacts selected as described in the previous sections, blacklisting all intra-chain contacts as they contained several orders of magnitude more information that could disturb the assignment of inter-chain edges (**Fig D in S1 Appendix**). The positioning of inter-chain contacts that we learned is shown in **Fig E in S1 Appendix**. Notably, this contact map shows far fewer residues with multiple contacts, suggesting that BN helped to resolve ambiguous cases with indirect correlation as expected. An example of conditional probability matrix for α_101_ with both same chain parent α_55_ and an inter-chain interaction with β_48_ is given in **Fig F in S1 Appendix**.

**Fig 4:**
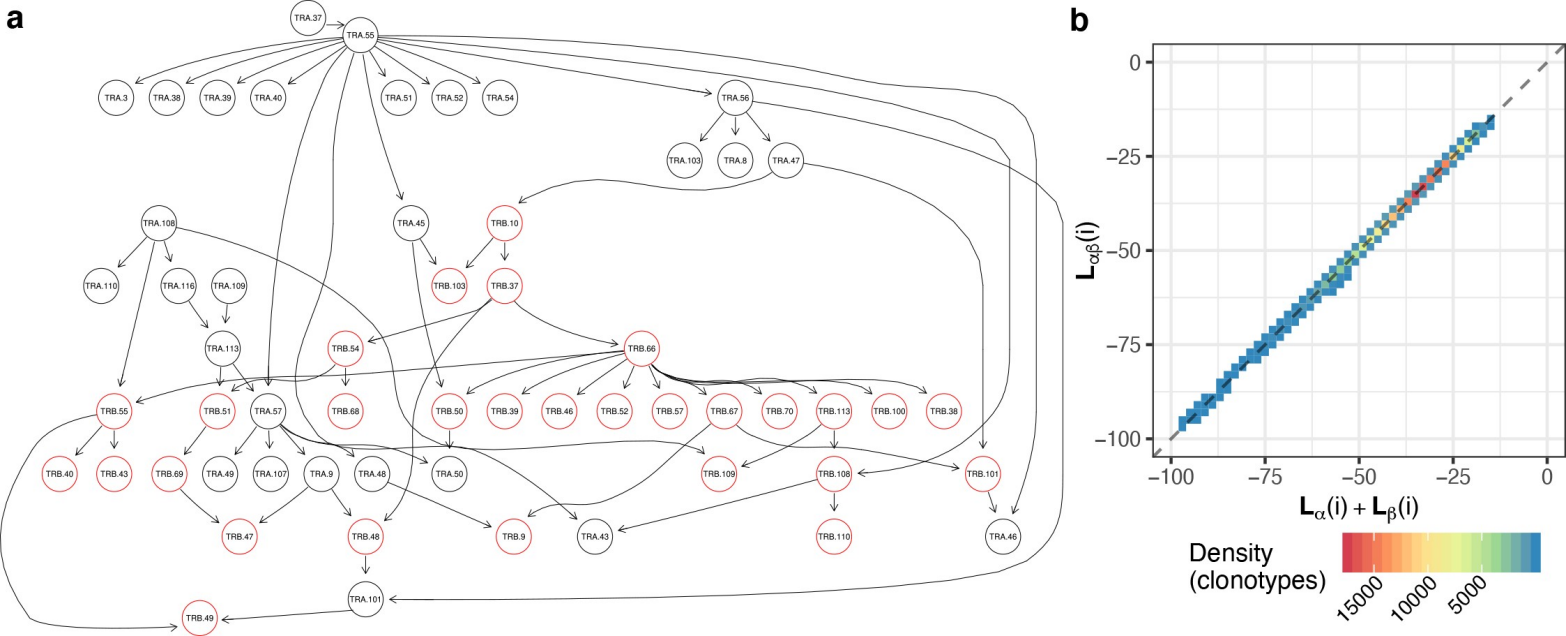
Bayesian network (BN) of TCRαβ complex residues. **a.** The graph of BN built with separately learned inter-chain contacts (shown in **S4 Fig**) whitelisted and residues that are not contacting according to contact frequency thresholding blacklisted. **b.** A density plot showing correlation between log-likelihood (LL) of BN for paired chains (y axis, computed using the network in **a.**) and sum of LLs of individual α and β chains for i^th^ clonotype from PairSEQ dataset. In order to compute individual chain LLs two independent networks were built by removing inter-chain edges and separating α and β residue components of the BN.

The resulting network shown in **Fig 4A** that was built by whitelisting all edges learned in the previous section featured more inter-chain edges than were obtained when learning the network as-is from input data with no constraints. There is, however, little if any difference in the likelihood of TCRαβ complexes compared to the sum of likelihoods from separate chains as shown in **Fig 4B**. Notably, small likelihood values in this figure stemmed from the fact that certain V/J gene combinations are quite rare in the PairSEQ dataset with frequency less than 10^−4^ and that the network did not include all possible edges between intra-chain residues which are in reality all interconnected, leading to the presence of lots of independent probability distribution products.

By computing the entropy based on all allowed amino acid profiles of the selected residues one can observe the following: the entropy of separate α chain is H_α_ = 4.68, separate β chain is H_β_ = 2.71, and the entropy of TCRαβ complexes is H_αβ_ = 7.43. The difference between these values is H_αβ_-H_α_-H_β_ = 0.04, suggesting that the amount of information conveyed by constraints imposed by α and β chain sequences on chain pairing is negligibly small.

### Amino acids at contact residues influence the conformation of the TCRαβ complex

**Fig 5:**
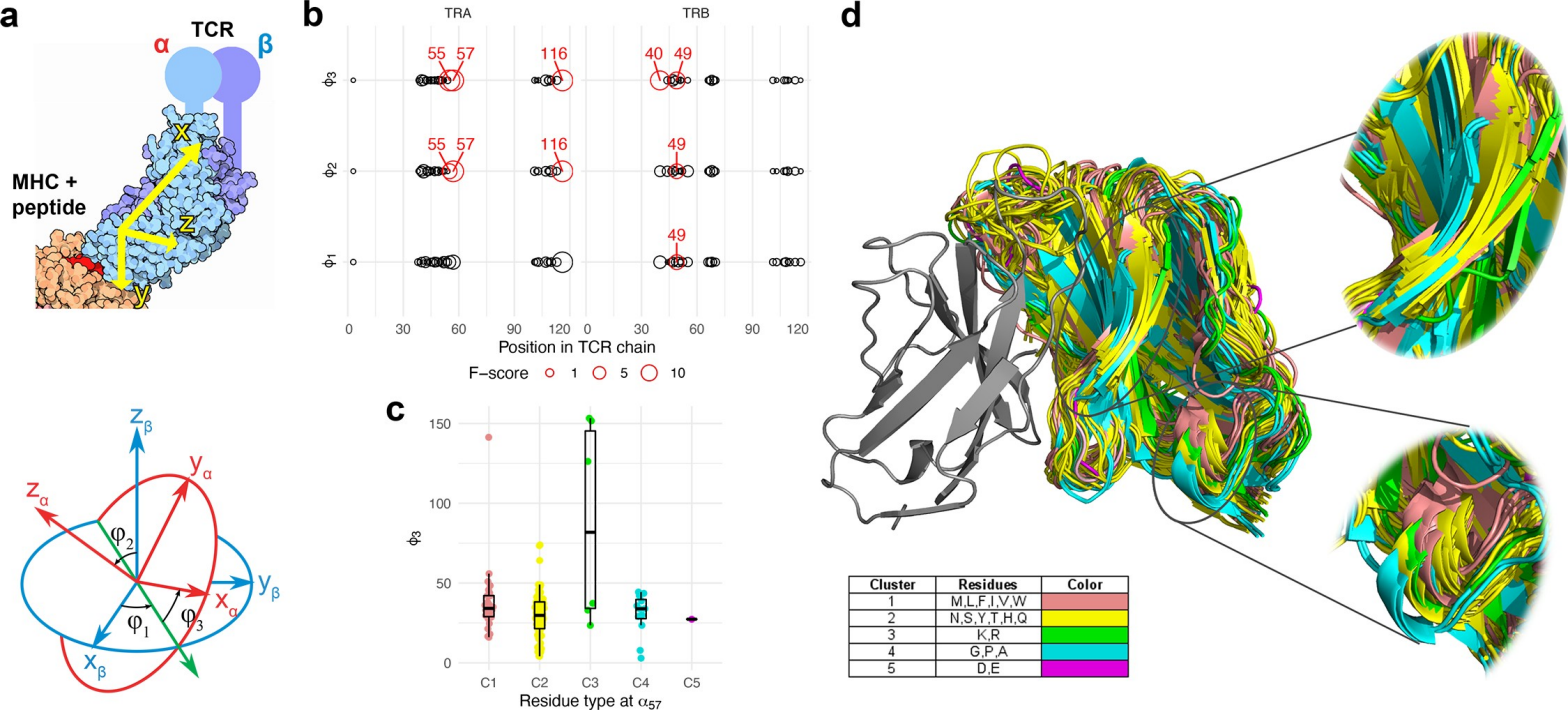
Contacting residues define mutual orientation of chains in TCRαβ complex. **a.** A schematic definition of angles between α and β chains. Principal axes (x_α_, y_α_, z_α_) and (x_β_, y_β_, z_β_) of both TCR chains are computed using the inertia tensor of all atoms of a given chain with the exception of constant domain atoms (**top panel**); representative orientation of principal axes in real TCR:pMHC complex are shown. Euler angles φ_1,2,3_ are then computed by superimposing chain centers of mass and computing angles between α and β principal axes (**bottom panel**). Illustrations were adapted from Wikimedia Commons (https://commons.wikimedia.org/wiki/File:63-T-CellReceptor-MHC.tif by David Goodsell and https://commons.wikimedia.org/wiki/File:Eulerangles.svg by Lionel Brits). **b.** Testing association between amino acid type (see Methods section and panel **d.** insert for amino acid cluster definition) and inter-chain angles. Point size shows ANOVA F-score for association between amino acid type and each of three Euler angles across TCR alpha and beta chain positions. The testing is performed for a non-redundant set of TCR chain orientations: all PDB structures with the same VαJαVβJβ are collapsed into a single observation with mean φ_1_, φ_2_ and φ_3_ angles to prevent biases from several complexes with the same TCR. Red circles and labels show contact positions where a significant association between amino acid content and inter-chain angle is present, determined as P < 0.05 (adjusted for multiple testing). **c.** Representative distribution of φ_3_ angle values for each amino acid type at α_57_ position. **d.** Visualization of all PDB structures aligned to a single representative TCR beta chain. TCR alpha chains are colored according to amino acid type at α_57_ position.

Given little pairing information encoded in contact residues we suggested that they may still play a role in defining the overall conformation of the TCRαβ complex. We therefore checked mutual orientation of α and β chains of complexes having distinct amino acids at some of the critical contact residues identified in the previous section and shown in **Fig E in S1 Appendix**. In order to define chain orientation, we first computed principal axes of chain atoms that provide a general description of the characteristics of a rigid body approximating a given chain (**Fig 5A top**), an approach that was previously successfully applied to describe the geometry of both TCR and antibody complexes [11]. We then used these axes to compute Euler angles between α and β chains that provide a comprehensive description of their mutual orientation (**Fig 5A bottom**).

In order to demonstrate that certain amino acid incidence at contacting residues can significantly alter TCRαβ complex conformation we performed a statistical analysis of association between inter-chain angles and contacting residue type. We defined 5 amino acid classes (see Methods section and **Fig G in S1 Appendix**) and performed an ANOVA test for association between three inter-chain Euler angles and amino acid class for human TCR:pMHC complexes from PDB database. Note that PDB database contains many redundant structures and the presence of multiple copies of the same TCR and antigen with little or no differences in their sequence may bias the analysis (**Fig H in S1 Appendix**). We therefore reduced the list of available TCR structures to a set of non-redundant VαJαVβJβ combinations (**Fig 5B**). Our results reveal several contacting residues that are associated with mutual orientation of TCR chains, for example the α_57_ residue (**Fig 5C**). Overlaying PDB structures using TCR beta chain as an anchor and coloring them based on the residue type at α_57_ position highlights differences in inter-chain for different residue types at this position as can be seen in **Fig 5D**. Of note, there may be potential combinatorial effects involving several contacting residues as depicted in **Fig I in S1 Appendix**, that are, however, hard to quantify due to limited number of distinct TCR structures available in PDB.

### Antigen-driven selection overrides pairing preferences

As overall differences in pairing preferences in TCRαβ complex were relatively subtle, we hypothesized that the TCR repertoire can still show αβ pairing preferences when subject to perturbations, such as antigen-driven selection and expansion. We have therefore investigated pairing preferences in TCRs specific to certain epitopes extracted from the VDJdb database [12] (see **Materials and Methods** section).

To study this case, we computed likelihood values of the observed TCRαβ complexes for the original dataset and datasets where shuffling was performed within and between epitope-specific TCR groups. The likelihoods were computed using a BN that solely included inter-chain contacts (**Fig D in S1 Appendix**) to minimize the V and J gene usage bias.

**Fig 6:**
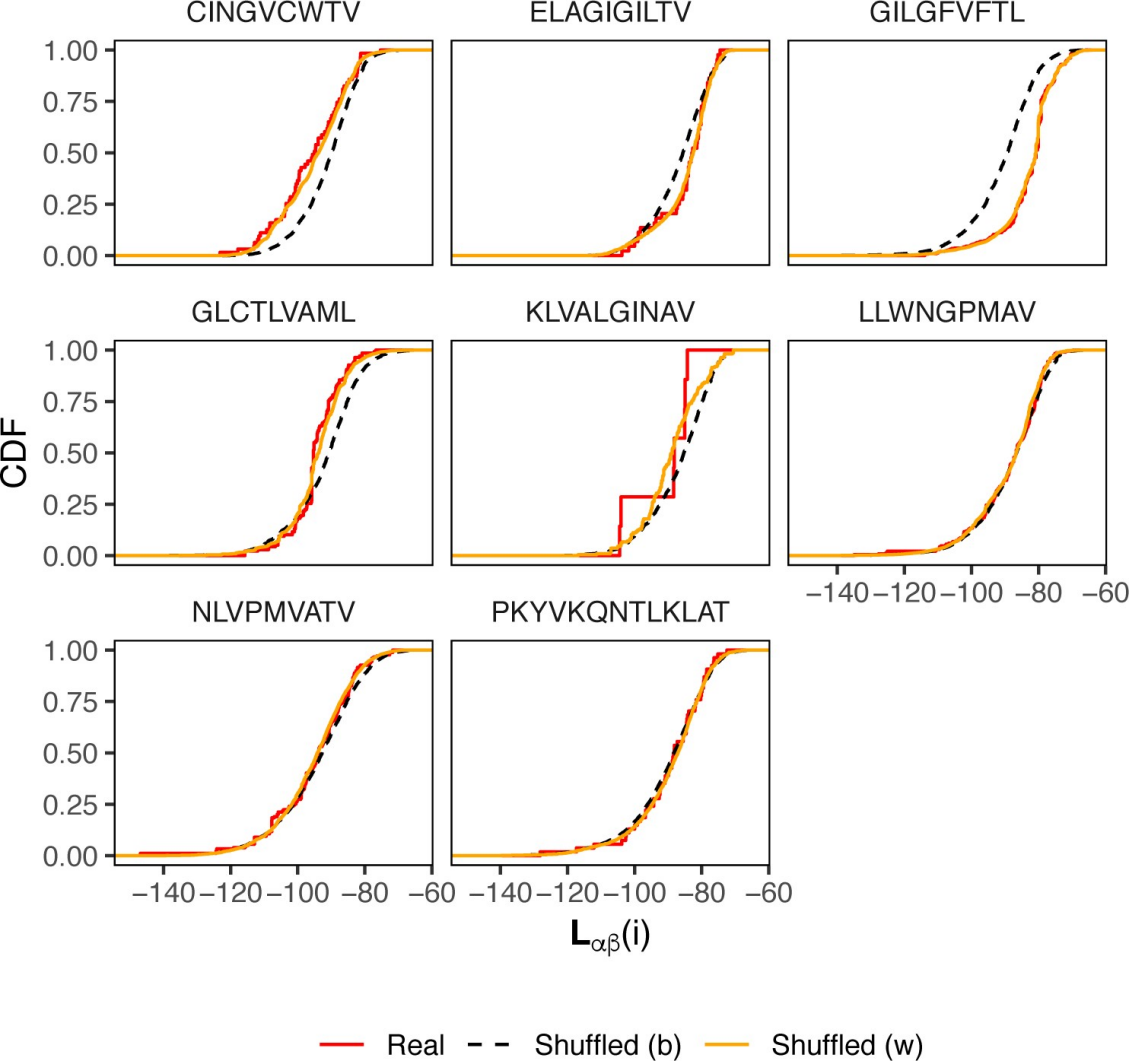
Log-likelihood (LL) distributions for TCRαβ pairs with known antigen specificity. Cumulative distribution functions of αβ pair LLs computed according to the model shown in **Fig 4A**. The plot shows n = 1388 real TCRαβ pairs from the VDJdb database (red curves), as well as pairs shuffled within groups specific to the same epitope (orange curves) and pairs shuffled across the entire dataset (dashed black curve). For the latter case, n = 10,000 pairs were selected at random for each epitope pair with re-sampling allowed in order to balance the dataset. Labels above panels are the cognate epitope sequences. Significant differences (Kolmogorov-Smirnov test P-value less than 0.05) between real and shuffled distributions are observed for CINGVCWTV (Kolmogorov-Smirnov test D-value = 0.25, P = 6x10^-4^), ELAGIGILTV (D = 0.23, P = 2x10^-2^), GILGFVFTL (D = 0.45, P < 10^−15^) and GLCTLVAML epitopes (D = 0.26, P = 3x10^-8^).

As demonstrated in **Fig 6**, selection based on epitope preferences can distort pairing preferences, given a rise in both more likely and less likely TCRαβ complexes compared to random pairing. The latter can be attributed to the residual effects of a specific choice of V and J segments at one of the chains, e.g. predominant usage of TRBV19 for A*02 GILGFVFTL epitope. On the other hand, there is little difference in pairing preferences within epitope-specific TCR groups so that shuffling chain pairs doesn't change the likelihood distribution, and one would expect little constraints on pairing in TCRαβ complexes recognizing the same epitope. Overall, this demonstrates that TCRαβ pairing constraints are weak enough to allow any chain combination suitable to recognize a given antigen.

### TCRαβ pairing preferences of invariant T-cells

**Fig 7:**
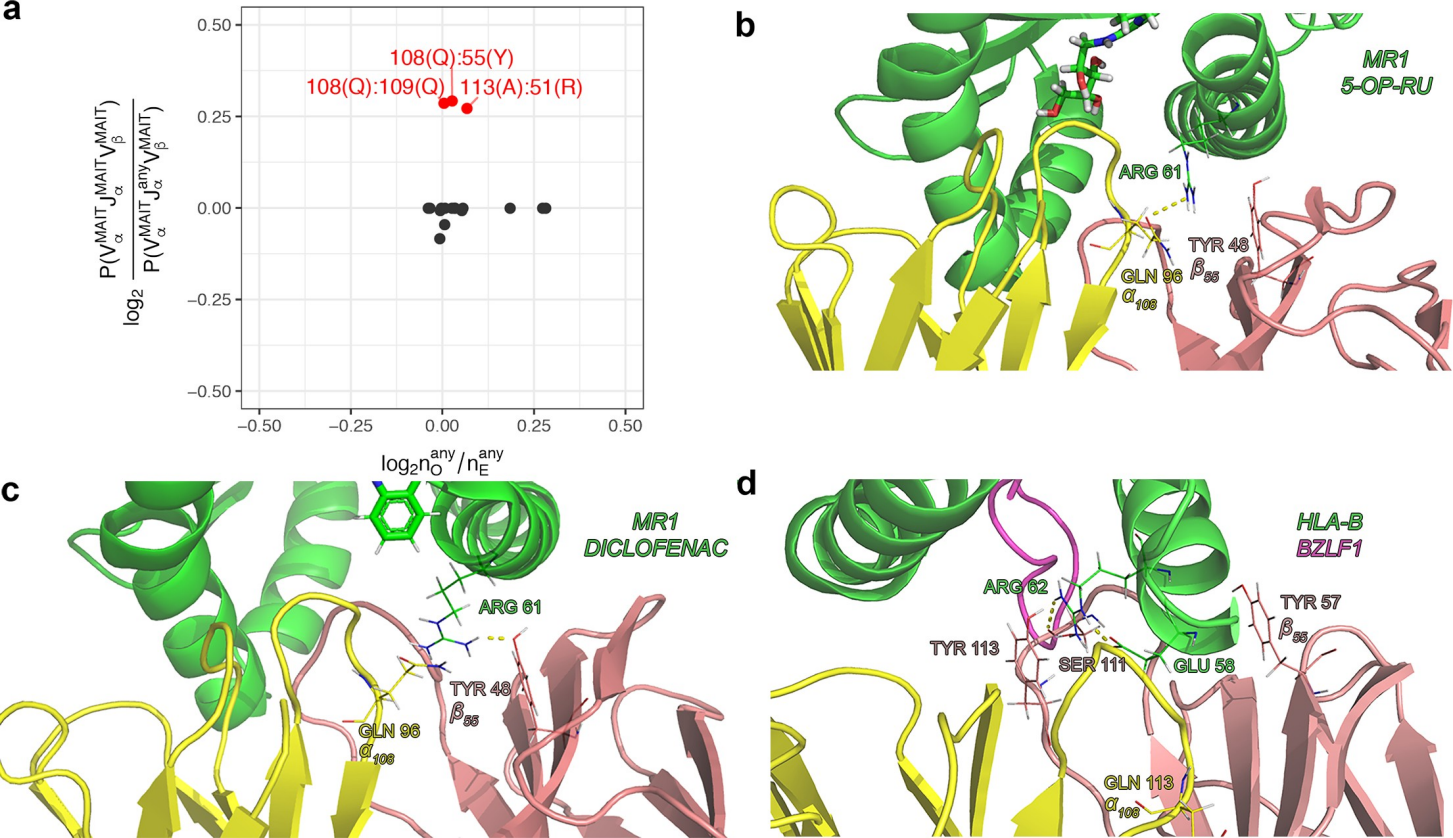
Characteristic residue contacts of MAIT TCRs. **a.** Scatter-plot of amino acid pair enrichment at contacting residues for the Jα gene choice of MAIT T-cells versus overall enrichment observed for given contact residues in the PairSEQ dataset. Y axis shows the log ratio of amino acid pair probabilities for VαJαVβ combinations corresponding to MAIT T-cells and those with a free choice for the Jα gene. X axis shows observed to expected amino acid pair count ratio at contacting residues in the PairSEQ dataset. Residue pairs with enriched amino acid pairs coming from MAIT Jα gene choice (y > 0.25, corresponding to ~19% increase in frequency) are colored in red and labeled. Not that overall enrichment for corresponding amino acid pairs in the PairSEQ dataset is relatively moderate (x < 0.125, corresponding to ~9% increase in frequency). **b.-d.** Structural data showing Glutamine (GLN, Q) at α_108_ and Tyrosine (TYR, Y) at β_55_ interacting with an Arginine (ARG) of MHC alpha-1 helix domain in the MAIT:MR1 complex. MR1 complex structures are shown in **b** (*4pj7*) and **c** (*5u1r*), **d** shows an non-MAIT TCR (having the same amino acids at α_108_ and β_55_) in complex with MHCI (*4jry*). Polar contacts between GLN:ARG and TYR:ARG are shown with dotted lines in **b** and **c**, but are absent in **d**. PDB structure chain coloring: green for MHC, yellow for TCRα and pink for TCRβ; antigen peptide in **d** is shown with purple.

As it is well-known that there are certain subsets of T-cells characterized by invariant T-cell receptor structure such as MAIT [13] and iNKT [14] cells, we decided to investigate selection biases related to T-cell specialization and phenotype. For example, MAIT cells were shown to be enriched in TCR sequences rearranged from TRAV1-2 and TRAJ12/20/33 that are mostly paired with TRBV6-4/20 [15]. As a validation of our framework, we investigated the enrichment of specific αβ contacts characteristic for MAIT cells using the PairSEQ dataset. We compared residue pair frequencies in VαJαVβ of MAIT cells with clones having MAIT VαVβ and any Jα choice (**Fig 7A**). We found several residue pairs that involve Jα and have higher amino acid pair frequency than expected by chance, however, little enrichment for these contacts in the whole dataset suggests that we have found indirect interactions driven by the need to recognize the MR1 molecule by MAIT cells rather than direct αβ contacts.

We turned to structural data on available MAIT TCR complexes to further investigate contacts enriched in MAIT subset, namely α_108_GLN and β_55_TYR. We visually inspected twenty available MAIT TCR-ligand-MR1 complex structures and a structure of conventional TCR having corresponding residues in Vβ and Jα obtained from the Protein Data Bank and used PyMOL to find contacts involving those residues. While we failed to detect any direct contact between α_108_ and β_55_, we found out that both of them can interact with ARG residue of the alpha-1 helix of the MR1 molecule: MR1 ARG is close to and faces both α_108_GLN and β_55_TYR.

In some of MAIT structures (PDB IDs *4pj7*, *4pj8* and *5d7j*) MR1 ARG is involved in a polar interaction with α_108_GLN only (**Fig 7B**), while in most of the remaining structures a polar interaction with β_55_TYR is observed (**Fig 7C**). At the same time, in the latter case ARG spatial orientation could be stabilized by the α_108_GLN side chain group from below even in the absence of a direct contact, so we suggest that both TCR residues are required to sandwich MR1 ARG. Notably, while there is a similarly placed ARG residue in the alpha-1 helix of a conventional MHCI molecule, it is situated far from corresponding TCR residues and doesn’t form any contacts with them (**Fig 7D**), so the interaction between MR1 ARG, α_108_GLN and β_55_TYR is likely a unique feature of MAIT TCRs.

To sum up, our inter-chain contact model did not predict any direct contact between α_108_ and β_55_ residues for conventional TCRs, which is in agreement with the available structural data, allowing us to spot and explore the enrichment of a specific residue pair characteristic for TCRs of MAIT cells.

### De-novo detection of invariant TCRs using αβ pairing biases

Results presented above support the hypothesis of unconstrained chain pairing in TCRαβ complex, and we observe pairing biases in cases of antigen-specific T-cell subsets and MAIT T-cells, but not at the level of entire T-cell repertoire. Thus, we hypothesize that deeper exploration of pairing biases can be a useful instrument for T-cell subset discovery. To demonstrate the feasibility of this method, we analyzed frequencies of VαJαVβJβ gene combinations in the PairSEQ dataset in order to detect cases of biased αβ pairing and explored the potential phenotype of corresponding T-cells using literature and published scRNASeq data.

**Fig 8:**
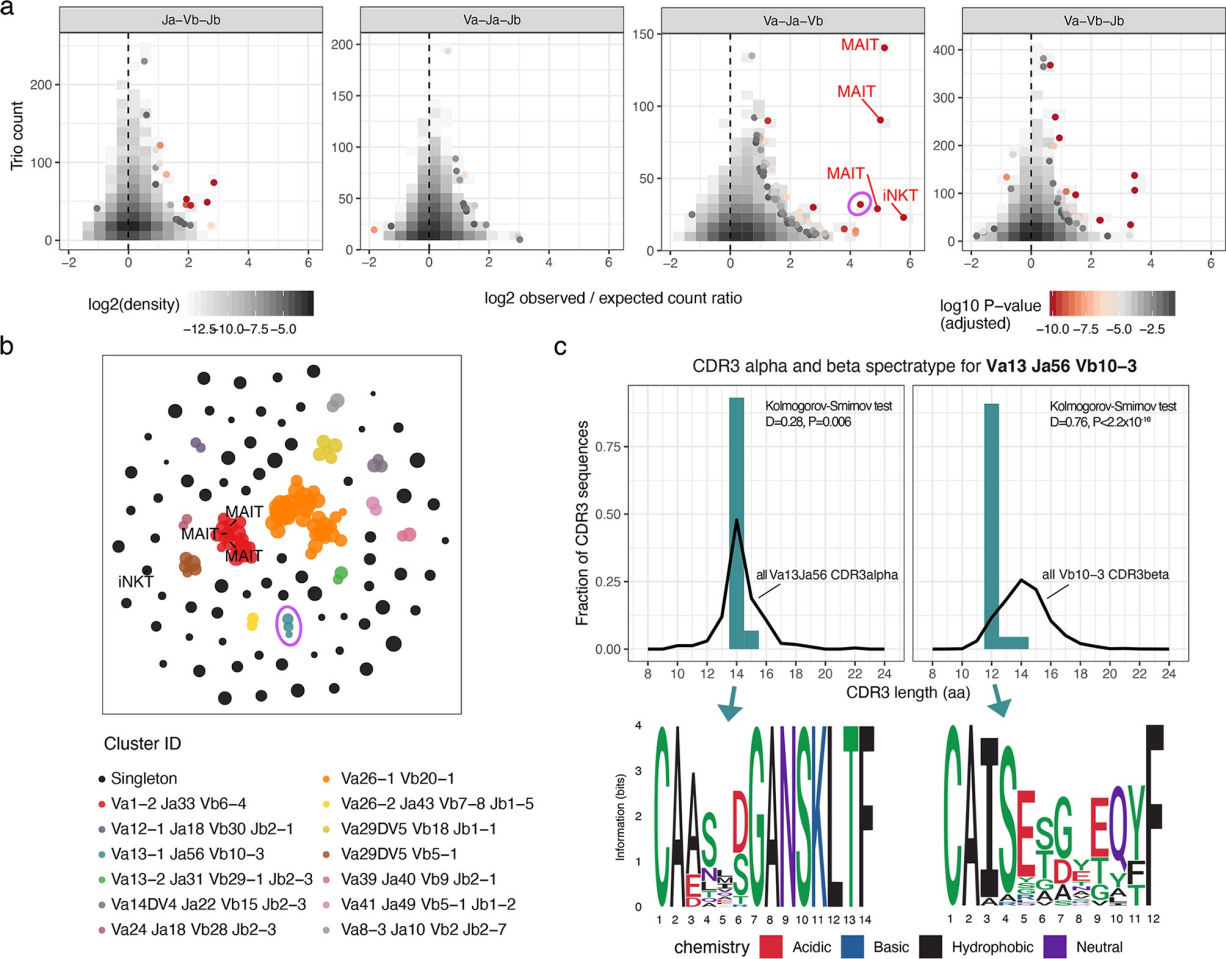
Exploring invariant TCR using enrichment analysis of VαJαVβJβ gene combinations. **a.** Scatterplot showing enrichment of certain TCR gene trios (unique combinations of three of four TCR germline genes, either JαVβJβ, VαVβJβ, VαJαJβ or VαJαVβ) in the PairSEQ dataset. Logarithm of the ratio of the observed and expected counts for all possible gene trios is plotted against their observed count. Expected count is calculated under the assumption of random αβ pairing as (count of α part alone) x (count of β part) / (total number of reads). Points are colored by the P-value of the hypergeometric enrichment test for the co-occurrence of α and β parts of the gene trio (adjusted for multiple comparisons using Holm method). Canonical MAIT (TRAV1-2, TRAJ12/20/33, TRBV6-4) and iNKT (TRAV10, TRAJ18, TRBV25-1) variants are highlighted with corresponding labels. Only gene trios supported by at least 10 reads are shown. Pink circle highlights the Va13 Ja56 Vb10-3 population. **b.** Grouping of selected TCR gene trios (having adjusted P < 0.05 for enrichment test and represented by at least 10 reads) according to overlap between their VαJαVβJβ gene sets. The plot shows the layout of the resulting graph of gene trios (nodes), having edges connecting pairs of nodes with exactly matching gene sets (missing genes, e.g. Vα in JαVβJβ, are considered as wildcards). Nodes of the graph are represented by points and are colored according to the connected component (cluster) of the network they were assigned to. Cluster ID is a combination of most frequent gene names in co-clustered trios. **c.** CDR3 spectratyping and motifs for the Va13 Ja56 Vb10-3 population. Top plots show distribution of CDR3 alpha (left) and beta (right) chains of the population compared to all PairSEQ TCRs rearranged with corresponding alpha or beta segments, note that only a single dominant length is present for both alpha and beta. Bottom plots show sequence logos of corresponding CDR3 lengths in the population.

We based our analysis on TCR gene trios, i.e. JαVβJβ, VαVβJβ, VαJαJβ and VαJαVβ combinations. The rationale behind this is that invariant TCRs of MAIT and iNKT cells are commonly defined based on the VαJαVβ gene trio [16]. We next compared the number of times each TCR gene combination is observed in the PairSEQ dataset and its count expected under the assumptions of random αβ pairing using hypergeometric enrichment test (**Fig 8A)**. As can be seen in the figure, conventional MAIT and iNKT cells are clear outliers marking several highly enriched VαJαVβ combinations. Notably, we also observed a high number or enriched TCR gene trios that are of unknown origin and were not described previously (**Table A in S1 Appendix**).

Grouping enriched TCR gene trios based on partial overlap between gene sets yields a number of large connected components, one of which is clearly linked to MAIT, and a singleton representing iNKT cells (**Fig 8B**). The Va13 Ja56 Vb10-3 population demonstrates a remarkable enrichment (pink circle in **Fig 8A and 8B, Table A in S1 Appendix**) that is close to MAIT and iNKT cells in magnitude. Further investigation of this population showed that it indeed features invariant TCRs that are dominated by 14aa and 12aa CDR3 alpha and beta sequences encoding a prominent motif (**Fig 8C**). We hypothesize that this simple approach based on quantification of biases in TCRαβ pairing can be applied to detect antigen-driven selection in TCR repertoire and, in some cases, provide a way to detect enriched subsets of unconventional T-cells.

## Discussion

In the present study we performed a deep and comprehensive analysis of T-cell receptor sequencing and structural data aimed at detecting biases in TCRα and β chain pairing preferences. We were able to identify dozens of TCR residues that are involved in inter-chain contacts, however, even those positions of either chain that can feature several amino acids corresponding to distinct V and J genes were relatively uniform in amino acid pair preference. Most of irregularities we detected were related to cases present in less than 100 cells per 10^5^ T-cells analyzed which, together with information measurement based on inferred residue probability distribution, provides a solid proof for nearly random pairing of TCRα and β chains. The consequence of our finding is that human T-cell repertoire in general feature almost any combination of TCR α and β chains in a given cell. This observation is in line with one of the aims of the adaptive immune system, which is to field the most diverse repertoire of T-cells in order to be able to successfully combat newly encountered pathogens.

One of the limitations of analysis is that it was aimed at germline parts of TCR as it is almost impossible to summarize the impact of the central part of CDR3 region that is mostly involved in antigen recognition and is hard to bring to a uniform indexing due to a huge variety of potential conformations of the resulting loop. We checked potential associations between various CDR3 features and failed to find any substantial correlations between values for alpha and beta chain CDR3 regions (**Fig J in S1 Appendix**).

Specific choice of contacting amino acids can nevertheless influence the overall conformation of the TCRαβ complex. As mutual orientation of chains affected by the choice of specific germline-encoded residues of V and J genes, knowing the possible spectrum of residues that affect conformation and corresponding orientations can aid in de-novo TCR:peptide:MHC complex reconstruction [17]. As only a handful of Vα/Jα/Vβ/Jβ allele combinations are currently present in the set of PDB structures, predictive models aimed at specific contacting residues can greatly extend the number of templates available for this task.

As expected, pairing in TCRαβ complexes can be heavily distorted by T-cell selection. Our results suggest that the selection of TCRs based on antigen specificity can lead to both more and less frequent αβ pairs depending on the antigen, suggesting that antigenic stimuli can override the uniform landscape of αβ pairing. Notably, chain pairing within the T-cell subset specific to the same epitope appears to be almost random, which is in line with our previous observations [18] and suggests that there is little competition between α and β chains in epitope recognition.

Our observation of random pairing in TCRαβ complexes in the absence of T-cell selection and the fact that there are specific T-cell subsets characterized by invariant TCR sequences lead us to explore pairing preferences in these subsets. Our results show that there is no intrinsic bias for interaction between α and β chains in invariant MAIT cells: observed pairing preferences stem from the need of a cooperative interaction between TCR α and β chains and the MR1 molecule recognized by MAIT cells. Furthermore, we were able to link observed irregularities in α and β chain pairing to previously described invariant T-cell subsets and detect novel homologous TCR sets, suggesting that our results can serve as a basis for detection of specialized T-cell subsets.

There are some open questions not discussed in the study, such as whether inter-chain interactions in TCRαβ complex can lead to distinct configurations that are more or less preferable for targeting specific epitopes. The methodology in this manuscript can be also extended to account for interaction between germline parts of the TCRαβ and MHC molecules, potentially leading to models describing docking and binding preferences in the TCRαβ:MHC complex.

## Materials and methods

### TCR:pMHC structural data analysis

We manually queried Protein Data Bank [10] for TCR:peptide:MHC complex structures. In total, 170 structures for both human and mouse were downloaded. All these structures were further cleaned up using the PyMOL software (Schrödinger, LLC) as follows: only one copy of each of the chains forming the complex (TCRα, TCRβ, peptide, MHCα and MHCβ/β2microglobulin) was left and all auxiliary proteins in case of several models; ions and ligands were deleted. After cleanup all structures were spatially superimposed according to the TCRα-TCRβ chain pair pose.

In order to summarize contact frequency at TCR α and β chain residues, we have set up a global indexing of residues across all possible V-J rearrangements.

IMGT numbering [10] was used for the V gene residue indexing, with Cys anchor residue of the CDR3 having an index of 104^th^ and preceding residues indexed according to IMGT alignment with gaps for a given V allele. We have included Cys104 and the following 3 residues (105–107) of V germline part of CDR3 and last 4 residues (108–111) of the J germline part, thus 111^th^ residue in our indexing system is the Phe/Trp anchor of J gene (part of the ‘[FW]GXG’ motif). We included 4 flanking CDR3 residues in the indexing based on our observation that they have almost fixed spatial position in CDR3 loop and rarely contact the antigen.

Uniform indexing is necessary for assessing association between amino acids at various positions of alpha and beta chains. We also computed the informativeness of all residues (positions) in aligned V and J genes as *I = 1 –H[p] / log(20)*, where *H* is the entropy function and *p* is the vector of amino acid probabilities at a given position (including ‘.’ for missing residues a.k.a. IMGT gaps).

Contacting residues between TCR α and β chains were selected if they have C_α_ atom distance closer than 15Å. We have also required a direct contact with a distance less than 5Å between closest atoms to be present for a given residue index pair (according to IMGT-based indexing introduced in previous section) for it to be considered in the analysis.

In order to perform an unbiased analysis of angles between TCR α and β chains (**Fig 5**) we removed structures having identical or nearly identical CDR3 region sequences leaving 106 structures in total.

### PairSEQ αβ repertoire sequencing data

Data from a paired TCRαβ receptor sequencing experiment (hereafter termed PairSEQ) was obtained from the previously published study of Howie *et al*. [5]. We have pooled datasets from ‘Experiment 1–5’ provided with the study. The dataset was converted to FASTA format preserving TCR pairing information and processed by the MIXCR software to produce V and J gene assignments and CDR3 nucleotide and amino acid sequence calls.

For some of our analysis focused on VαJαVβJβ assignments, we have summarized the data according to V and J gene call scores. We weighted each of the potential V-J assignments based on their score, for example, if we detect a TRBV-X with MIXCR match score of 200.0 and TRBV-Y with a score of 100.0 coupled with TRBJ-Z supported by 3 distinct clonotypes (defined as unique combination of V, J and CDR3 nucleotide sequence), we split it into two distinct V-J segment combinations: TRBV-X:TRBJ-Z with a frequency of 2 and TRBV-Y:TRBJ-Z with a frequency of 1. The weighting is performed in order to resolve ambiguous cases, especially cases when several TRBV or TRAV genes are tied due to the short read length of PairSEQ data: in this case, if selecting the first assignment in a non-randomized manner, one cannot rule out amino acid sequence biases for V gene part not covered by sequencing. The total number of unique PairSEQ clonotypes analyzed for computing VαJαVβJβ combination frequencies is ~3x10^5^.

### Bayes network analysis

We used BNLearn R package [11] to infer Bayes Networks of amino acid probability distributions at TCR α and β chain residues. A hill-climbing (*hc*) and a hybrid algorithm (*rsmax2*) was used for the network construction with an Akaike Information Criterion (AIC) score as an objective. AIC score represents a trade-off between the goodness of fit of the model and the simplicity, which is the number of residue-residue interactions in our case. After fitting to the data, inferred Bayes Networks were used for scoring TCRαβ complexes based on computed Log Likelihood (LL) values for instances. LL was computed as the logarithm of the probability of observing a given set of TCR α and β chain residues according to the fitted probabilistic model using either unconditional probabilities of amino acids at a given position or conditional probabilities in case certain residues are found to be dependent (interacting) based on Bayes Network structure.

We have also heavily relied on the ability to both whitelist and blacklist an edge in order to decouple intra- and inter-chain contact networks as described in corresponding Results section. Running Bayes Network analysis for the entire set of TCR residues from both chains leads to a network that mostly recaptures linkage between residues within the same chain, ignoring inter-chain contacts that appear to be far less informative. While inter-chain interactions may be significantly enriched in frequency than expected, their magnitude (i.e. the amount of information they confer) appears to be too small for them to be included into the network according to AIC score optimization when intra-chain interactions are also considered (AIC discourages less informative interactions to balance between network complexity and informativeness). In order to capture those informative inter-chain contacts, we ran analysis in two steps: during the first step we considered only intra-chain contacts, while at the second step we ignored intra-chain contacts and focused on inter-chain ones by constructing a Bayes Network with only inter-chain contacts allowed.

### Specific TCR data analysis

Data on T-cell receptor sequences with known antigen specificity was downloaded from the VDJdb database (vdjdb.cdr3.net) and filtered to include only records where both α and β chain sequences are known. Only antigens with at least 30 paired-chain records were selected for further analysis. The resulting epitope set is the following: CINGVCWTV (n = 76 paired sequences), ELAGIGILTV (44), GILGFVFTL (603), GLCTLVAML (148), KLVALGINAV (32), LLWNGPMAV (239), NLVPMVATV (187), PKYVKQNTLKLAT (59). The exact version of the VDJdb database used for the analysis is stored together with source code and other datasets.

## Supporting information

S1 Appendix Containing Supplementary Figures and Tables (Fig A-I and Table A). (PDF)
