# Supplementary material for: Comprehensive analysis of structural and sequencing data reveals almost unconstrained chain pairing in TCRαβ complex
Source: PLoS Comput Biol. 2020 Mar 12;16(3):e1007714. doi: 10.1371/journal.pcbi.1007714 (PMC7093030; doi:10.1371/journal.pcbi.1007714)
Supplement: S1 Appendix — (PDF) [file pcbi.1007714.s001.pdf]

Supplementary Figures and Tables (S1 Appendix)

Fig A. Amino acid pairing statistics at contacting residues. A. Scatter-plot of χ^2 statistic values versus mutual information for AA pair frequency matrices of each contacting residue pair. **b.** Mutual information is negatively correlated with conservation (information content) of V and J germline residues. **c.** Scatterplot of residue pair contact frequencies and mutual information of AA pair frequency matrices.

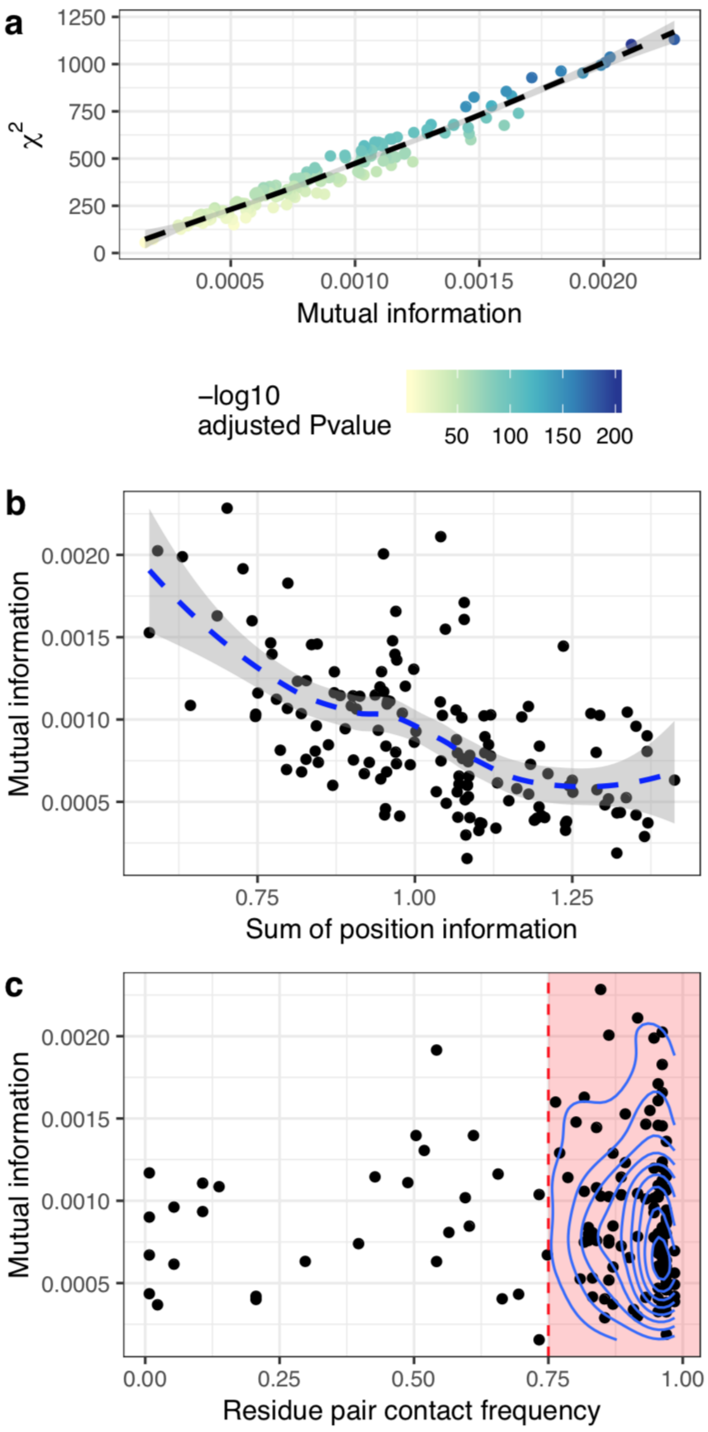

Fig B. A case of an indirect correlation between residue amino acid profiles. a. Contact frequency, χ^2 and mutual information for various TCRα residues contacting β_{101}. Note that α_{47} is contacted ~95% of times, while α_{43} is only contacted at a rate of ~11%. **b.** Tables of observed to expected contact count ratios showing highly non-random profiles for Histidine in α_{43} and Tryptophan in α_{47}. The correlation between these two table columns is R ≈ 1. **c.** Co-occurrence of amino acids between α_{43} and α_{47} in the set of human V genes. Numbers indicates the quantity of V genes with a given combination. The gene having Histidine and Tryptophan in corresponding positions is highlighted with a red box.

a

	TRA residue index	Contact frequency	ChiSQ	MI
1	47	0.95	928	0.0017
2	46	0.94	779	0.0015
3	43	0.11	580	0.0011
4	49	0.95	500	0.001
5	39	0.01	343	7e−04

b

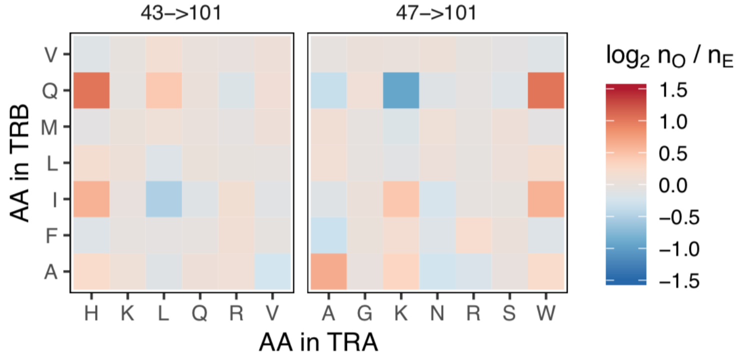

c

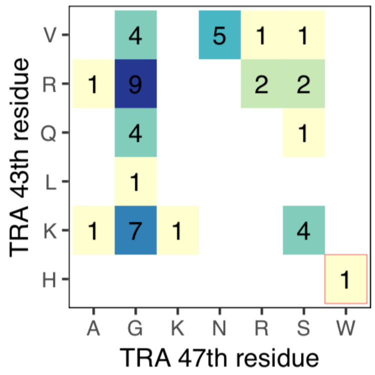

Fig C. Bayes network of residue amino acid profiles. The plot shows a graph encoding dependencies between amino acid profiles of all TCRαβ complex residues built for the entire PairSEQ dataset with no constraints on allowed and disallowed edges. Black and red nodes show TCRα and TCRβ residues respectively. The only inter-chain contacts inferred are $\beta_{108}\rightarrow\alpha_{37}$, and $\alpha_{46}\rightarrow\beta_{10}$.

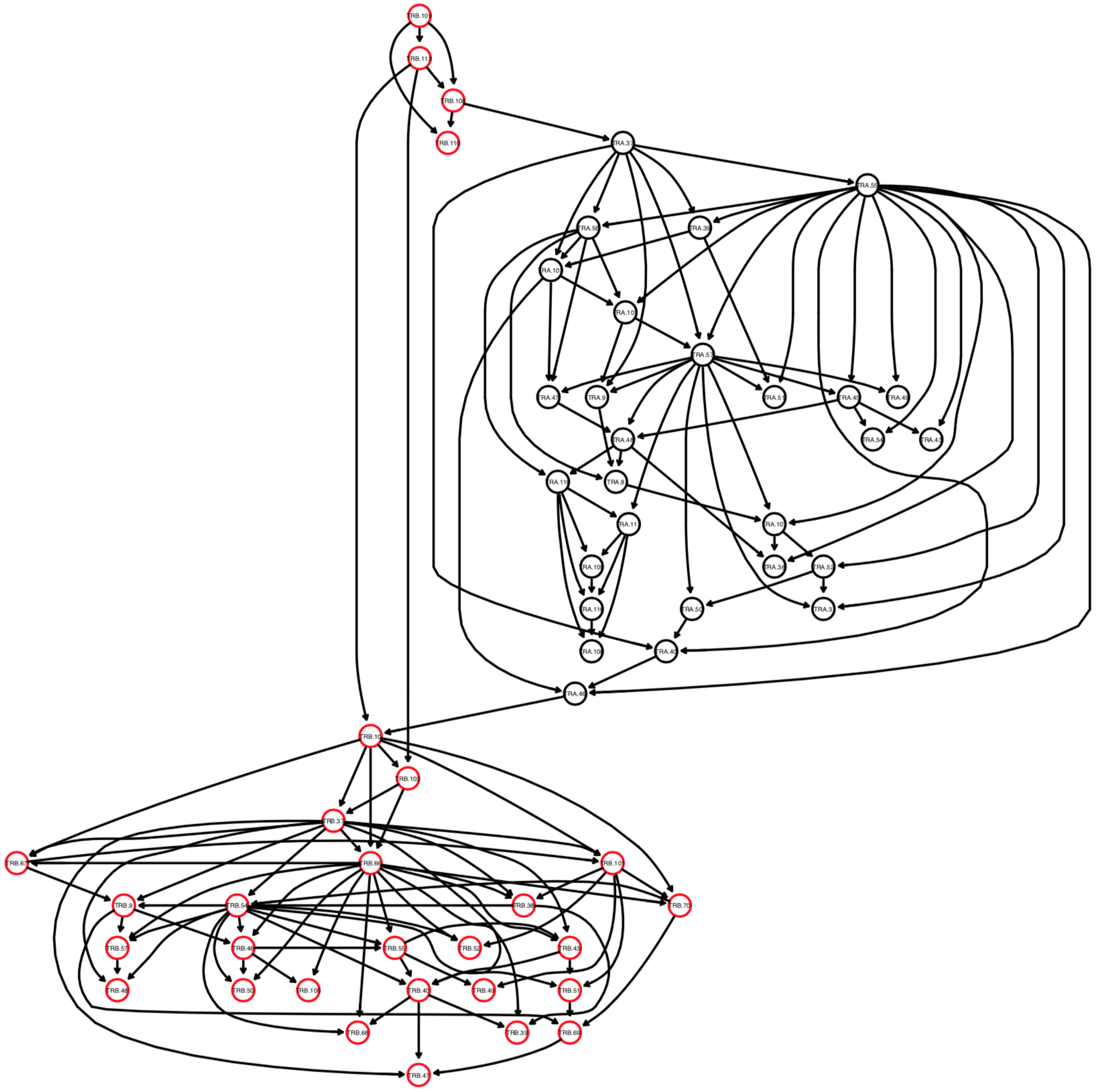

Fig D. Bayes network of inter-chain contacts. The plot shows a graph encoding dependencies between amino acid profiles of selected contacting residues built for the entire PairSEQ dataset with only inter-chain residue edges allowed. Black and red nodes show TCRα and TCRβ residues respectively.

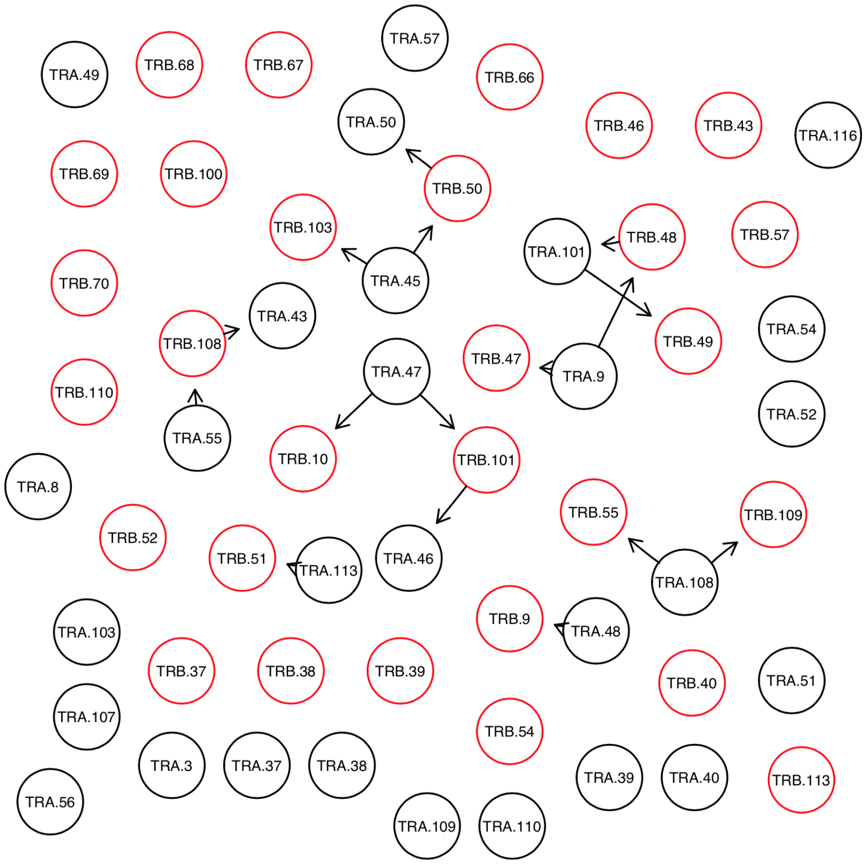

Fig E. Contact map with inferred direct αβ contacts. A contact map of TCRαβ complexes with contacting residue pairs inferred by Bayes network (BN) analysis shown in **Fig D** highlighted with labels. Greater (>) and less (<) signs show node directionality in BN, first and second numbers in node label represent α and β chain residue indices respectively. Node color shows mutual information of amino acid pair frequency matrices. Node size represents contact frequency for residue pairs inferred from structural data, grey nodes are the ones that fall below 0.75 contact frequency threshold.

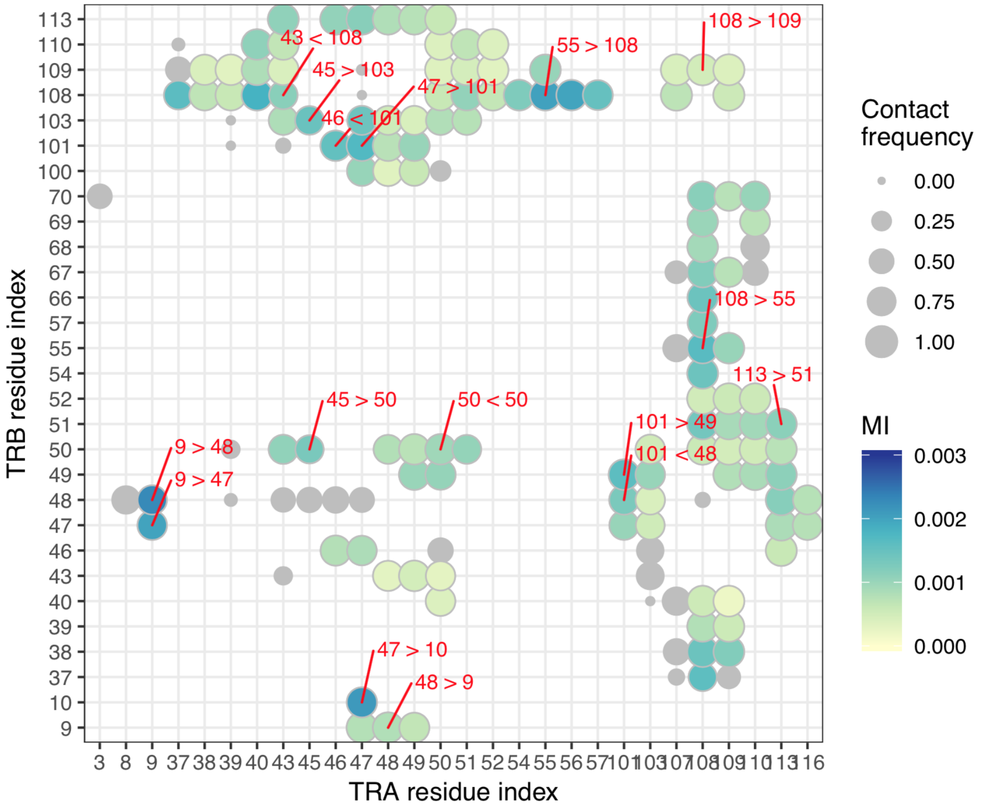

Fig F. Representative conditional probabilities learned using Bayes network. Conditional probabilities for residue α_{101} given residue α_{55} (green labels above panels) and residue β_{48} (orange labels). Subtle differences for e.g. Leucine (L) at α_{101} between Glycine (G) and Lysine (K) at β_{48} given Isoleucine (I) at α_{55} can be noted.

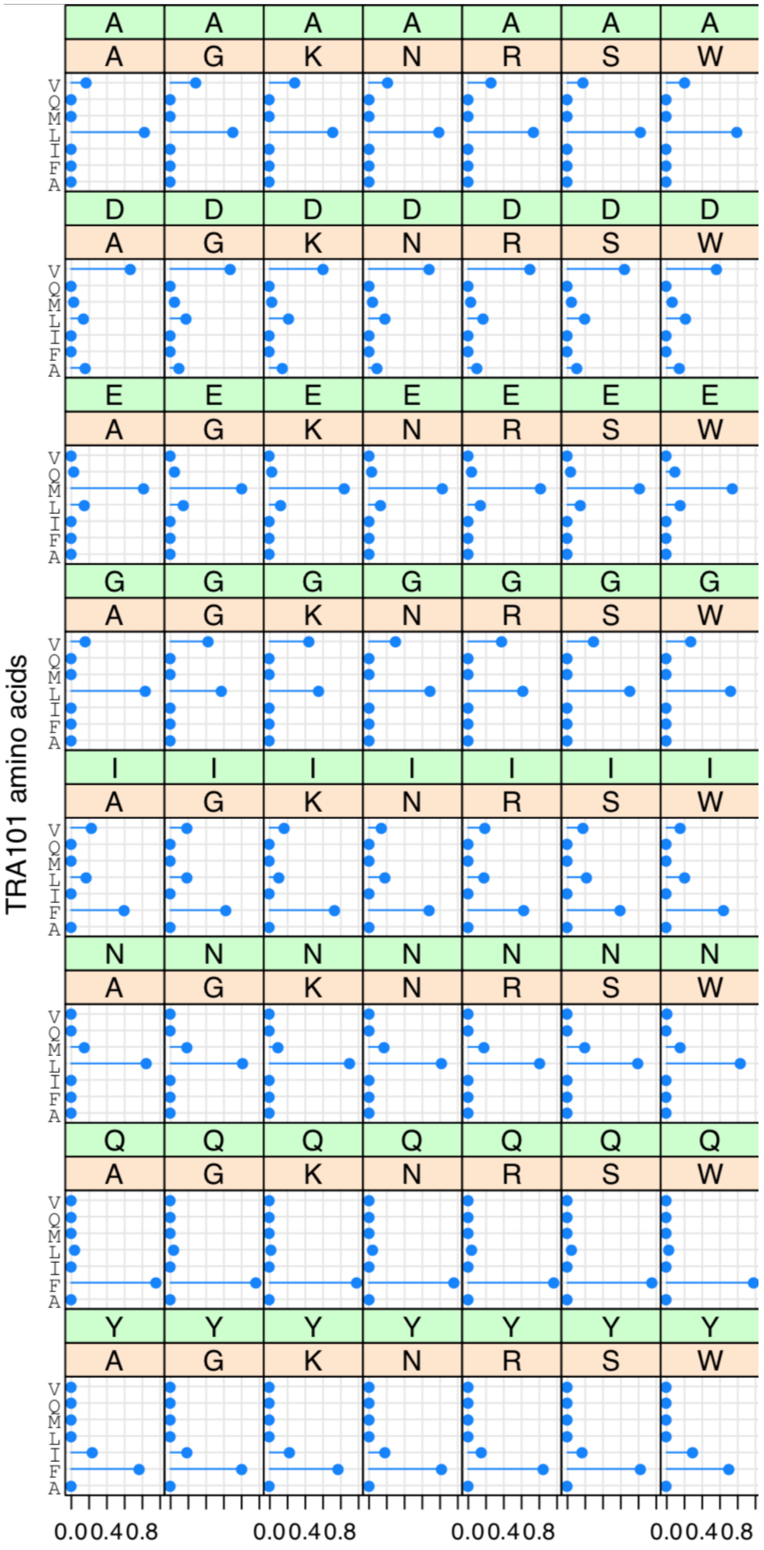

Fig G. Classifying amino acids based on their physicochemical features. Using PCA to perform placement and clustering of amino acids based on their physicochemical properties. K-means clustering with k=5 was performed to separate amino acids into 5 distinct groups.

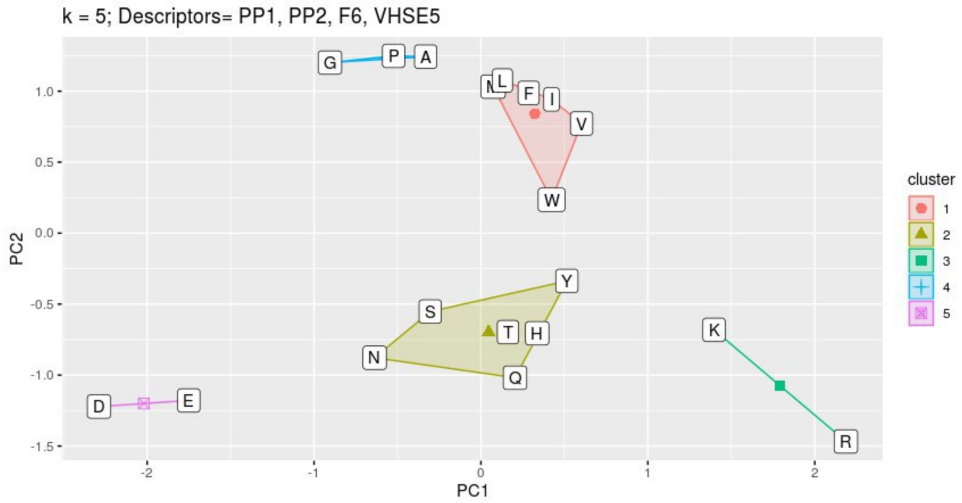

Fig H. Exploring Euler angles between TCR alpha and beta chains. a. A schematic definition of angles between α and β chains. Principal axes ($x\alpha$, $y\alpha$, $z\alpha$) and ($x\beta$, $y\beta$, $z\beta$) of both TCR chains are computed using the inertia tensor of all atoms of a given chain with the exception of constant domain atoms (top panel); representative orientation of principal axes in real TCR:pMHC complex are shown. Euler angles $\varphi_{1,2,3}$ are then computed by superimposing chain centers of mass and computing angles between α and β principal axes (bottom panel). Illustrations were adapted from Wikimedia Commons (https://commons.wikimedia.org/wiki/File:63-T-CellReceptor-MHC.tif by David Goodsell and https://commons.wikimedia.org/wiki/File:Eulerangles.svg by Lionel Brits). **b.** and **c.** - scatter plots of φ_1 and φ_3 angles between α and β chains in complexes having $\alpha_{50} \rightarrow \beta_{50}$ and $\alpha_{108} \rightarrow \beta_{55}$ contacts respectively. Note the differences in orientation between chains of TCRαβ complexes: difference in φ_1 angle between chains for complexes having either Proline (P) or other residue at β_{50} (**b**), and difference in φ_3 angle for complexes having either Isoleucine (I) or Asparagine (N) at α_{108} (**c**). The (**b**) panel shows that the presence of Proline at β_{50} residue substantially restricts the available φ_1 angle value range: 8° vs 44° on average for Proline versus any other residue ($P < 10^{-8}$, two-tailed T-test). On the other hand, panel (**c**) demonstrates that the choice of either Isoleucine or Asparagine at α_{108} residue leads to substantially different φ_3 angle values, 11° and 31° on average respectively ($P < 10^{-3}$, two-tailed T-test). However, these observations are severely biased by the presence of structures with redundant TCRs in the PDB dataset: for complexes shown in (**b**) panel 7/8 complexes with N at α_{108} are TRAV27/TRAJ42/TRBV19/TRBJ2-7 and 7/9 complexes with I at α_{108} are TRAV21/TRAJ6/TRBV6-5/TRBJ2-2. This redundancy can explain highly significant P-values when comparing inter-chain angles.

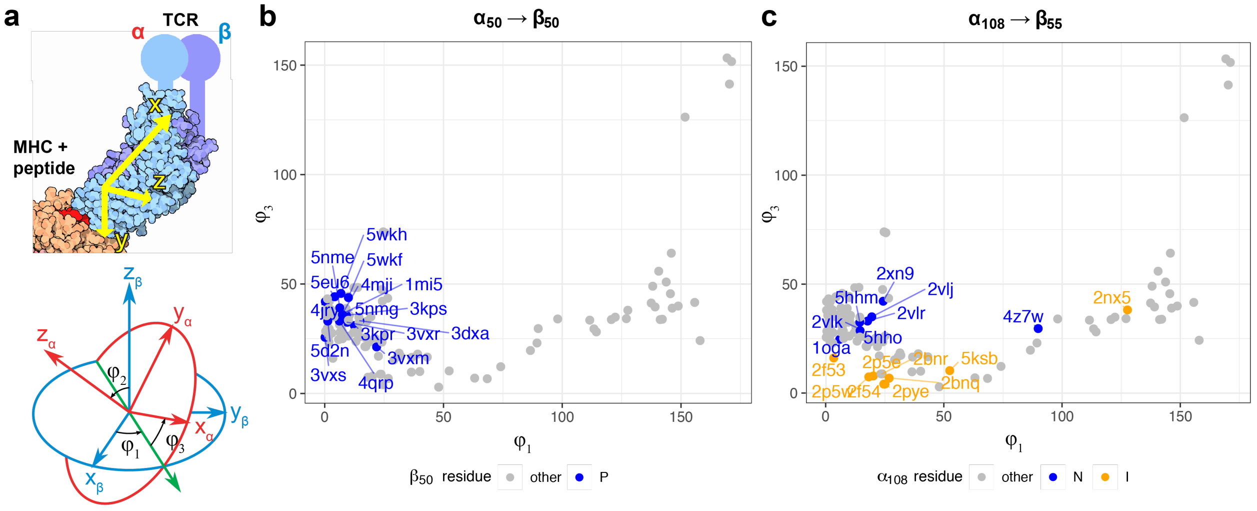

Fig I. Details of inter-chain contacts that influence mutual orientation of TCR alpha and beta chains. a. Contacting residues at alpha chain positions 40, 42, 55 and 57 (IMGT numbering). Note that different residues are forming inter-chain contacts in representative TCR:pMHC structures. **b.** Similar to **Fig 5d**, but colored by a combination of amino acid types at TCR alpha residues #42 and #57, highlighting potential combinatorial effect from several TCR alpha residues.

a.

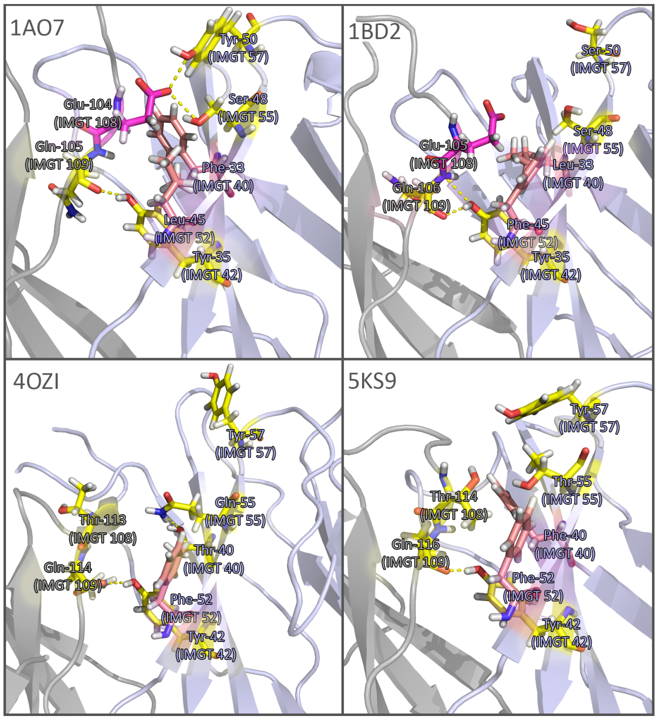

b.

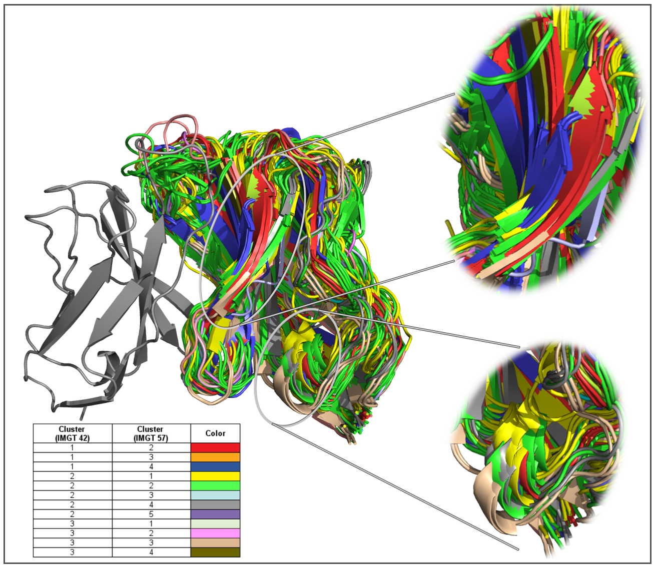

Fig J. Correlation analysis of CDR3 features of paired TCR alpha and beta chains. CDR3 length and means of Kidera factors (KF1-10) are shown. Pearson correlation coefficients between alpha and beta chain values and corresponding Bonferroni-adjusted P-values are provided above each subplot.

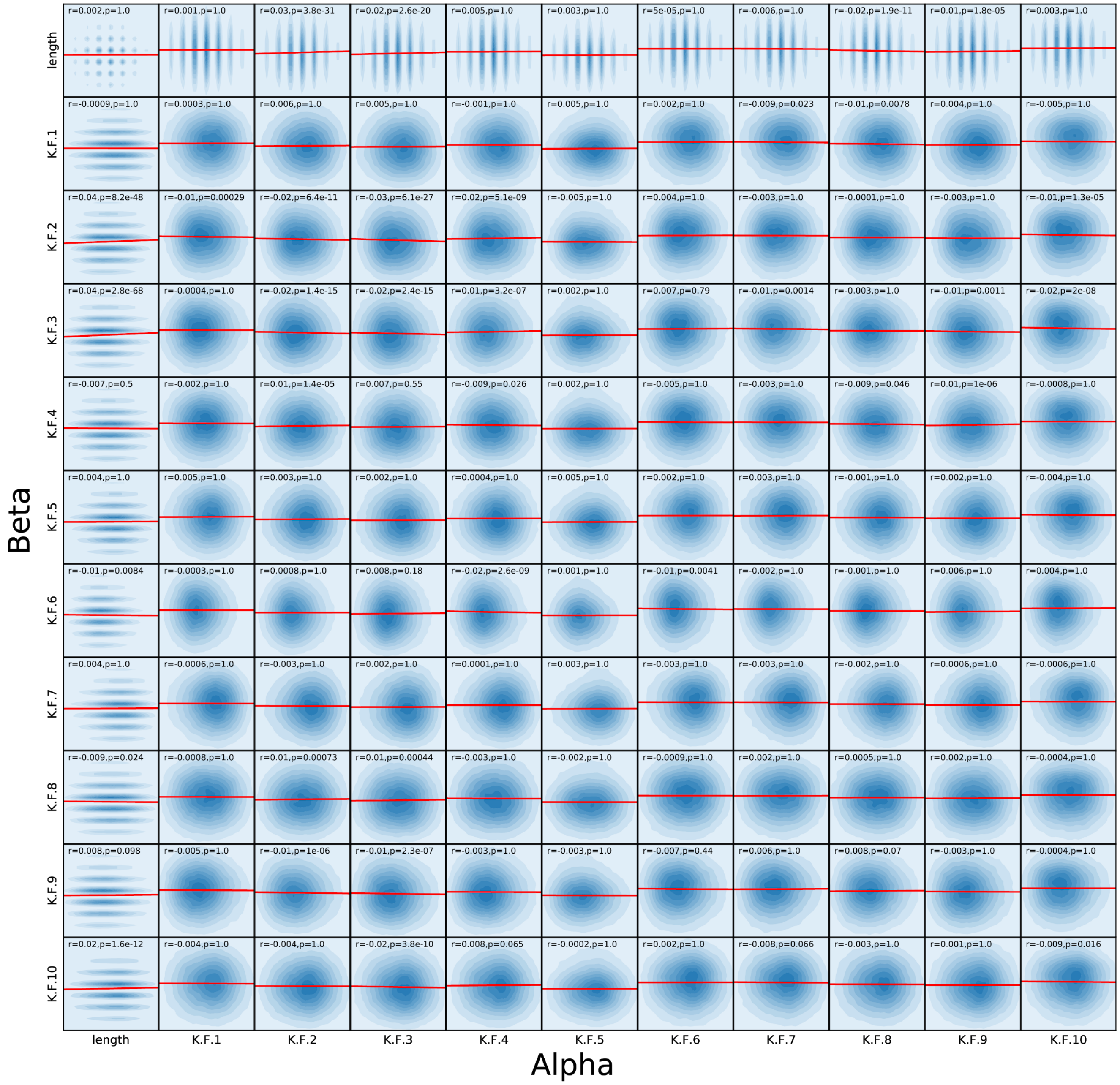

Table A. Table of enriched V/J gene trios. Cluster ID specifies groups of trios that have overlapping genes. Known subset specifies canonical MAIT or iNKT rearrangements. Observed (obs) and expected (exp, given random alpha-beta pairing) counts are shown for the PairSEQ dataset. A multiple testing-adjusted P-value of a hypergeometric enrichment test is provided for each gene trio.

Cluster ID	Va	Ja	Vb	Jb	Known subset	Count (obs)	Count (exp)	Adjusted P-value
Va8-3 Ja10 Vb2 Jb2-7	X	TRAJ10	TRBV2	TRBJ2-7		85	35	4.3E-08
Va8-3 Ja10 Vb2 Jb2-7	TRAV8-3	TRAJ10	TRBV2	X		45	15	1.4E-05
Va41 Ja49 Vb5-1 Jb1-2	TRAV41	TRAJ49	TRBV5-1	X		60	26	7.3E-05
Va41 Ja49 Vb5-1 Jb1-2	TRAV41	X	TRBV5-1	TRBJ1-2		43	18	1.2E-02
Va39 Ja40 Vb9 Jb2-1	TRAV39	X	TRBV9	TRBJ2-1		72	32	3.7E-05
Va39 Ja40 Vb9 Jb2-1	TRAV39	TRAJ40	TRBV9	X		31	10	4.4E-04
Va29DV5 Vb5-1	TRAV29DV5	TRAJ39	TRBV5-1	X		56	21	4.3E-06
Va29DV5 Vb5-1	TRAV29DV5	TRAJ6	TRBV5-1	X		22	4	5.1E-06
Va29DV5 Vb5-1	TRAV29DV5	X	TRBV5-1	TRBJ1-5		55	24	7.6E-04
Va29DV5 Vb5-1	TRAV29DV5	X	TRBV5-1	TRBJ2-1		168	110	1.1E-02
Va29DV5 Vb5-1	TRAV29DV5	TRAJ52	TRBV5-1	X		52	24	1.7E-02
Va29DV5 Vb18 Jb1-1	TRAV29DV5	X	TRBV18	TRBJ1-5		66	26	5.1E-07
Va29DV5 Vb18 Jb1-1	TRAV29DV5	X	TRBV18	TRBJ1-1		89	44	3.3E-05
Va29DV5 Vb18 Jb1-1	TRAV29DV5	X	TRBV18	TRBJ1-2		70	31	1.0E-04
Va29DV5 Vb18 Jb1-1	TRAV29DV5	TRAJ24	TRBV18	X		19	5	5.8E-03
Va29DV5 Vb18 Jb1-1	TRAV29DV5	X	TRBV18	TRBJ1-6		40	17	1.9E-02
Va26-2 Ja43 Vb7-8 Jb1-5	TRAV26-2	X	TRBV7-8	TRBJ1-5		38	13	1.5E-03
Va26-2 Ja43 Vb7-8 Jb1-5	TRAV26-2	TRAJ43	TRBV7-8	X		27	8	2.6E-03
Va26-1 Vb20-1	TRAV12-3	X	TRBV20-1	TRBJ2-3		260	149	0.0E+00
Va26-1 Vb20-1	TRAV26-1	X	TRBV20-1	TRBJ1-1		216	113	0.0E+00
Va26-1 Vb20-1	TRAV26-1	X	TRBV20-1	TRBJ1-3		97	35	0.0E+00
Va26-1 Vb20-1	TRAV26-1	X	TRBV20-1	TRBJ2-1		368	237	1.6E-11
Va26-1 Vb20-1	TRAV13-1	TRAJ17	TRBV20-1	X		90	38	1.6E-10
Va26-1 Vb20-1	X	TRAJ10	TRBV20-1	TRBJ1-4		46	12	9.6E-10
Va26-1 Vb20-1	X	TRAJ17	TRBV20-1	TRBJ1-2		122	58	6.4E-09
Va26-1 Vb20-1	TRAV12-3	TRAJ18	TRBV6-4	X		14	1	9.5E-09
Va26-1 Vb20-1	TRAV26-1	X	TRBV20-1	TRBJ1-2		199	118	1.4E-07
Va26-1 Vb20-1	TRAV12-3	TRAJ17	TRBV20-1	X		75	33	5.7E-07
Va26-1 Vb20-1	X	TRAJ3	TRBV20-1	TRBJ1-1		99	48	4.5E-06
Va26-1 Vb20-1	TRAV26-1	TRAJ49	TRBV20-1	X		94	48	1.1E-05
Va26-1 Vb20-1	X	TRAJ29	TRBV20-1	TRBJ1-1		116	61	1.3E-05
Va26-1 Vb20-1	TRAV12-3	TRAJ48	TRBV20-1	X		62	26	1.5E-05
Va26-1 Vb20-1	TRAV9-2	TRAJ23	TRBV20-1	X		98	52	2.6E-05
Va26-1 Vb20-1	TRAV26-1	TRAJ23	TRBV20-1	X		98	52	4.2E-05
Va26-1 Vb20-1	TRAV12-3	X	TRBV20-1	TRBJ2-5		200	128	7.3E-05

Va26-1 Vb20-1	TRAV12-3	TRAJ41	TRBV20-1	X		38	14	1.0E-04
Va26-1 Vb20-1	TRAV12-3	TRAJ10	TRBV20-1	X		77	38	1.4E-04
Va26-1 Vb20-1	TRAV12-3	TRAJ23	TRBV20-1	X		79	40	2.9E-04
Va26-1 Vb20-1	TRAV4	TRAJ15	TRBV20-1	X		40	15	4.9E-04
Va26-1 Vb20-1	TRAV6	TRAJ23	TRBV20-1	X		58	27	6.3E-04
Va26-1 Vb20-1	TRAV26-1	TRAJ30	TRBV20-1	X		70	35	1.2E-03
Va26-1 Vb20-1	X	TRAJ24	TRBV20-1	TRBJ2-5		94	50	1.2E-03
Va26-1 Vb20-1	TRAV13-1	X	TRBV20-1	TRBJ2-1		382	288	2.4E-03
Va26-1 Vb20-1	X	TRAJ23	TRBV20-1	TRBJ2-1		230	159	2.8E-03
Va26-1 Vb20-1	TRAV26-1	TRAJ29	TRBV20-1	X		80	43	4.6E-03
Va26-1 Vb20-1	TRAV26-1	X	TRBV20-1	TRBJ2-7		365	276	7.0E-03
Va26-1 Vb20-1	TRAV26-1	TRAJ39	TRBV20-1	X		92	53	7.2E-03
Va26-1 Vb20-1	TRAV12-3	TRAJ18	X	TRBJ2-3		40	17	7.8E-03
Va26-1 Vb20-1	TRAV26-1	TRAJ43	TRBV20-1	X		77	42	1.1E-02
Va26-1 Vb20-1	X	TRAJ15	TRBV20-1	TRBJ2-1		161	106	2.0E-02
Va26-1 Vb20-1	TRAV12-3	TRAJ11	TRBV20-1	X		55	27	2.9E-02
Va26-1 Vb20-1	TRAV26-1	TRAJ41	TRBV20-1	X		57	29	3.0E-02
Va26-1 Vb20-1	TRAV26-1	TRAJ34	TRBV20-1	X		75	41	4.2E-02
Va26-1 Vb20-1	TRAV1-1	TRAJ24	X	TRBJ2-5		38	16	4.6E-02
Va24 Ja18 Vb28 Jb2-3	TRAV24	TRAJ18	TRBV28	X		21	4	2.1E-05
Va24 Ja18 Vb28 Jb2-3	TRAV24	TRAJ18	X	TRBJ2-3		25	9	3.1E-02
Va14DV4 Ja22 Vb15 Jb2-3	TRAV14DV4	TRAJ22	TRBV15	X		30	4	0.0E+00
Va14DV4 Ja22 Vb15 Jb2-3	TRAV14DV4	X	TRBV15	TRBJ2-3		52	17	1.1E-07
Va14DV4 Ja22 Vb15 Jb2-3	TRAV14DV4	TRAJ22	X	TRBJ2-3		73	32	6.7E-07
Va13-2 Ja31 Vb29-1 Jb2-3	TRAV13-2	TRAJ31	TRBV29-1	X		25	6	2.2E-05
Va13-2 Ja31 Vb29-1 Jb2-3	TRAV13-2	X	TRBV29-1	TRBJ2-3		59	29	3.6E-02
Va13-1 Ja56 Vb10-3	TRAV13-1	TRAJ56	TRBV10-3	X		32	2	0.0E+00
Va13-1 Ja56 Vb10-3	TRAV13-2	TRAJ56	TRBV10-3	X		12	1	1.8E-04
Va13-1 Ja56 Vb10-3	X	TRAJ56	TRBV10-3	TRBJ2-7		24	7	2.7E-02
Va12-1 Ja18 Vb30 Jb2-1	TRAV12-1	TRAJ18	TRBV30	X		22	4	9.5E-05
Va12-1 Ja18 Vb30 Jb2-1	X	TRAJ18	TRBV30	TRBJ2-1		27	9	3.2E-02
Va1-2 Ja33 Vb6-4	TRAV1-2	TRAJ12	TRBV6-4	X	MAIT	29	1	0.0E+00
Va1-2 Ja33 Vb6-4	TRAV1-2	TRAJ20	TRBV6-4	X	MAIT	91	3	0.0E+00
Va1-2 Ja33 Vb6-4	TRAV1-2	TRAJ33	TRBV6-4	X	MAIT	141	4	0.0E+00
Va1-2 Ja33 Vb6-4	TRAV1-2	X	TRBV6-4	TRBJ2-1		138	13	0.0E+00
Va1-2 Ja33 Vb6-4	TRAV1-2	X	TRBV6-4	TRBJ2-2		35	3	0.0E+00
Va1-2 Ja33 Vb6-4	TRAV1-2	X	TRBV6-4	TRBJ2-3		107	10	0.0E+00
Va1-2 Ja33 Vb6-4	X	TRAJ20	TRBV6-4	TRBJ2-1		53	14	0.0E+00
Va1-2 Ja33 Vb6-4	X	TRAJ33	TRBV6-4	TRBJ2-1		74	10	0.0E+00
Va1-2 Ja33 Vb6-4	X	TRAJ33	TRBV6-4	TRBJ2-3		49	8	0.0E+00
Va1-2 Ja33 Vb6-4	X	TRAJ20	TRBV6-4	TRBJ2-3		45	11	3.3E-11

Va1-2 Ja33 Vb6-4	TRAV1-2	TRAJ41	TRBV6-4	X		12	1	3.1E-08
Va1-2 Ja33 Vb6-4	X	TRAJ33	TRBV6-4	TRBJ2-2		19	3	1.4E-06
Va1-2 Ja33 Vb6-4	TRAV1-2	TRAJ33	X	TRBJ2-1		194	125	2.3E-05
Va1-2 Ja33 Vb6-4	TRAV1-2	X	TRBV6-4	TRBJ2-6		12	1	2.9E-05
Va1-2 Ja33 Vb6-4	TRAV1-2	TRAJ33	X	TRBJ2-6		43	15	7.4E-05
Va1-2 Ja33 Vb6-4	TRAV1-2	TRAJ33	TRBV4-2	X		41	14	1.5E-04
Va1-2 Ja33 Vb6-4	TRAV1-2	TRAJ33	TRBV20-1	X		135	82	3.9E-04
Va1-2 Ja33 Vb6-4	TRAV1-2	TRAJ33	TRBV4-3	X		44	19	1.8E-02
Va1-2 Ja33 Vb6-4	TRAV1-2	X	TRBV4-2	TRBJ2-1		51	24	4.0E-02
Singleton	TRAV10	TRAJ18	TRBV25-1	X	iNKT	23	0	0.0E+00
Singleton	TRAV20	X	TRBV6-2	TRBJ1-1		44	9	0.0E+00
Singleton	TRAV20	X	TRBV6-3	TRBJ1-1		44	9	0.0E+00
Singleton	TRAV41	TRAJ50	TRBV2	X		15	1	1.6E-10
Singleton	TRAV21	X	TRBV27	TRBJ2-3		104	46	2.5E-09
Singleton	TRAV8-1	TRAJ34	TRBV28	X		37	10	2.8E-07
Singleton	TRAV8-3	TRAJ34	TRBV15	X		27	5	5.2E-07
Singleton	TRAV22	X	TRBV29-1	TRBJ2-7		50	17	1.4E-06
Singleton	TRAV20	TRAJ22	TRBV30	X		25	5	2.0E-06
Singleton	TRAV38-2DV8	TRAJ9	TRBV7-9	X		15	2	3.4E-06
Singleton	TRAV12-1	TRAJ48	TRBV24-1	X		20	3	4.6E-06
Singleton	TRAV26-2	X	TRBV27	TRBJ1-1		50	18	1.9E-05
Singleton	TRAV1-1	X	TRBV28	TRBJ2-7		101	52	4.3E-05
Singleton	TRAV13-2	TRAJ6	TRBV14	X		20	4	4.4E-05
Singleton	TRAV34	TRAJ26	X	TRBJ1-6		13	2	5.9E-05
Singleton	TRAV6	TRAJ16	TRBV20-1	X		40	15	7.2E-05
Singleton	TRAV14DV4	TRAJ28	TRBV9	X		33	10	7.7E-05
Singleton	TRAV39	TRAJ45	TRBV20-1	X		61	28	1.7E-04
Singleton	TRAV26-1	TRAJ28	TRBV9	X		36	12	3.3E-04
Singleton	X	TRAJ4	TRBV29-1	TRBJ2-5		46	17	4.8E-04
Singleton	TRAV8-3	TRAJ21	TRBV14	X		14	2	5.1E-04
Singleton	TRAV3	TRAJ42	TRBV30	X		22	5	7.3E-04
Singleton	TRAV25	X	TRBV19	TRBJ1-5		65	30	7.8E-04
Singleton	TRAV12-1	TRAJ17	TRBV2	X		30	9	9.2E-04
Singleton	TRAV2	TRAJ15	TRBV2	X		31	10	1.0E-03
Singleton	TRAV5	TRAJ34	TRBV28	X		35	12	1.1E-03
Singleton	TRAV35	TRAJ42	X	TRBJ1-2		68	34	1.1E-03
Singleton	TRAV25	TRAJ11	TRBV7-9	X		18	4	1.5E-03
Singleton	TRAV8-3	TRAJ8	TRBV28	X		36	13	1.6E-03
Singleton	TRAV12-1	TRAJ43	X	TRBJ1-1		89	48	1.8E-03
Singleton	TRAV29DV5	X	TRBV7-9	TRBJ1-2		89	48	2.0E-03
Singleton	TRAV13-2	TRAJ18	TRBV24-1	X		10	1	2.1E-03

Singleton	TRAV13-2	X	TRBV7-2	TRBJ1-5		57	26	2.3E-03
Singleton	TRAV8-3	TRAJ49	X	TRBJ2-4		24	7	2.6E-03
Singleton	TRAV9-2	TRAJ57	TRBV12-5	X		12	2	3.5E-03
Singleton	TRAV8-1	TRAJ6	TRBV30	X		20	5	3.7E-03
Singleton	TRAV10	TRAJ12	TRBV7-9	X		13	2	4.3E-03
Singleton	TRAV13-2	X	TRBV7-8	TRBJ1-5		52	23	4.3E-03
Singleton	TRAV41	X	TRBV14	TRBJ1-1		27	8	4.5E-03
Singleton	TRAV14DV4	TRAJ54	TRBV18	X		25	7	4.7E-03
Singleton	TRAV13-1	TRAJ30	TRBV7-3	X		17	4	6.1E-03
Singleton	TRAV36DV7	TRAJ23	X	TRBJ1-4		10	1	6.2E-03
Singleton	TRAV8-3	TRAJ20	X	TRBJ2-5		77	41	6.5E-03
Singleton	TRAV23DV6	TRAJ41	TRBV5-1	X		21	6	6.7E-03
Singleton	TRAV1-1	X	TRBV30	TRBJ2-7		48	21	7.7E-03
Singleton	X	TRAJ27	TRBV2	TRBJ2-4		19	5	8.3E-03
Singleton	X	TRAJ40	TRBV10-3	TRBJ1-5		27	9	8.6E-03
Singleton	X	TRAJ45	TRBV12-5	TRBJ2-1		25	8	8.7E-03
Singleton	TRAV14DV4	X	TRBV20-1	TRBJ1-5		121	73	8.7E-03
Singleton	TRAV25	X	TRBV27	TRBJ1-2		50	22	9.1E-03
Singleton	TRAV24	X	TRBV19	TRBJ2-7		50	22	9.6E-03
Singleton	X	TRAJ53	TRBV24-1	TRBJ1-5		24	7	1.1E-02
Singleton	TRAV6	TRAJ22	TRBV14	X		13	2	1.1E-02
Singleton	TRAV9-2	TRAJ53	TRBV9	X		55	25	1.1E-02
Singleton	TRAV4	TRAJ9	TRBV13	X		11	2	1.3E-02
Singleton	TRAV14DV4	TRAJ39	TRBV24-1	X		15	3	1.4E-02
Singleton	TRAV13-2	X	TRBV2	TRBJ1-5		64	32	1.5E-02
Singleton	TRAV22	TRAJ28	TRBV5-8	X		12	2	1.7E-02
Singleton	TRAV22	TRAJ28	TRBV5-4	X		12	2	1.7E-02
Singleton	TRAV27	TRAJ39	X	TRBJ1-2		45	20	2.2E-02
Singleton	X	TRAJ7	TRBV15	TRBJ2-7		21	6	2.4E-02
Singleton	TRAV12-1	TRAJ45	TRBV18	X		26	8	2.7E-02
Singleton	TRAV1-1	TRAJ13	X	TRBJ1-2		42	19	2.8E-02
Singleton	TRAV25	X	TRBV19	TRBJ2-1		81	44	2.9E-02
Singleton	TRAV29DV5	X	TRBV16	TRBJ1-1		11	2	3.1E-02
Singleton	TRAV41	TRAJ43	TRBV18	X		19	5	3.1E-02
Singleton	TRAV21	TRAJ18	TRBV7-3	X		11	2	3.4E-02
Singleton	X	TRAJ45	TRBV2	TRBJ2-1		72	38	3.4E-02
Singleton	TRAV8-2	X	TRBV14	TRBJ2-1		44	19	3.4E-02
Singleton	TRAV8-4	X	TRBV14	TRBJ2-1		44	19	3.5E-02
Singleton	TRAV12-2	TRAJ13	TRBV25-1	X		13	2	3.5E-02
Singleton	TRAV2	TRAJ13	TRBV28	X		37	15	4.1E-02
